# A comparison of methods for analyzing a binary composite endpoint with partially observed components in randomized controlled trials

**DOI:** 10.1002/sim.9203

**Published:** 2021-09-29

**Authors:** Tra My Pham, Ian R. White, Brennan C. Kahan, Tim P. Morris, Simon J. Stanworth, Gordon Forbes

**Affiliations:** 1 MRC Clinical Trials Unit at UCL, Institute of Clinical Trials and Methodology, London, UK; 2 NHS Blood & Transplant, Oxford University Hospitals and the University of Oxford, Oxford, UK; 3 Biostatistics & Health Informatics Department, King’s College London, London, UK

**Keywords:** compatibility, composite endpoints, missing data, multiple imputation, RCTs

## Abstract

Composite endpoints are commonly used to define primary outcomes in randomized controlled trials. A participant may be classified as meeting the endpoint if they experience an event in one or several components (eg, a favorable outcome based on a composite of being alive and attaining negative culture results in trials assessing tuberculosis treatments). Partially observed components that are not missing simultaneously complicate the analysis of the composite endpoint. An intuitive strategy frequently used in practice for handling missing values in the components is to derive the values of the composite endpoint from observed components when possible, and exclude from analysis participants whose composite endpoint cannot be derived. Alternatively, complete record analysis (CRA) (excluding participants with any missing components) or multiple imputation (MI) can be used. We compare a set of methods for analyzing a composite endpoint with partially observed components mathematically and by simulation, and apply these methods in a reanalysis of a published trial (TOPPS). We show that the derived composite endpoint can be missing not at random even when the components are missing completely at random. Consequently, the treatment effect estimated from the derived endpoint is biased while CRA results without the derived endpoint are valid. Missing at random mechanisms require MI of the components. We conclude that, although superficially attractive, deriving the composite endpoint from observed components should generally be avoided. Despite the potential risk of imputation model mis-specification, MI of missing components is the preferred approach in this study setting.

## 1 Introduction

Composite endpoints are commonly used to define primary outcomes in randomized controlled trials, such as those in rheumatoid arthritis, tuberculosis, and cardiovascular diseases.^1–5^ A composite endpoint can be constructed from two or more components. As a simple example of a composite endpoint, a participant may be classified as meeting the endpoint if they experience an event in one or several components; for instance, a favorable outcome in trials assessing tuberculosis treatments may be defined based on a composite endpoint of the participant being alive and attaining negative culture results during follow-up.

In practice, not all components of a composite endpoint are fully observed, and components that are not always missing or observed simultaneously complicate the analysis of the composite endpoint. A strategy often used in practice is to perform a complete record analysis (CRA) in which only participants with observed data in all components are included. Such a strategy may yield less efficient, and potentially even biased, estimates when the components are not missing completely at random (MCAR).

To make more use of available data, another strategy is to derive the composite endpoint from observed components when possible, and exclude from analysis participants whose composite endpoint cannot be derived.^6,7^ In the aforementioned example of trials assessing tuberculosis treatments, suppose that a participant is classified as having an unfavorable outcome if they either die or have positive culture results. For a given participant with missing culture results, their endpoint can be derived to be unfavorable if we know that they die before the end of the trial, whereas their endpoint cannot be ascertained (and therefore considered missing) if they are alive. Another type of composite endpoint is the time to the first of two or more events, whichever occurs first, and might be of primary interest in many clinical trials. For example, in cancer trials, a commonly used primary endpoint is progression-free survival, defined as the time from randomization to tumor progression or death. Some participants may be lost to follow-up before experiencing an event (ie, the progression component is missing), while their vital status at the end of the trial might be obtained from linkage to external death registry data (ie, the mortality component is “observed”). This setting was previously explored by Daniel and Tsiatis,^8^ who demonstrated how external information on the mortality component of the composite endpoint for participants lost to follow-up before experiencing an event can be incorporated in augmented inverse probability weighted estimating equations in order to increase efficiency.

Previously, O’Keeffe et al^9^ studied a binary composite endpoint with seven components, measured repeatedly for individuals during follow-up. The authors investigated the scenario in which if one component of the composite endpoint is missing at a particular time point, then all components are missing. Thus, it would not be possible to derive the value of the composite endpoint at time points where the components are missing. Rombach et al^10^ focused on composite endpoints that are linear functions of the components, which generally cannot be derived if at least one component is missing. Nevertheless, some scoring manuals allow for a small number of components to be substituted by the mean score of the available components (ie, single imputation with the average of the observed values).

While an analysis of the derived endpoint (i) is intuitively sensible, since we sometimes can determine a participant’s endpoint from the value of only one component, and (ii) uses more observed data compared with a CRA, it is not clear under which missingness mechanisms of the components valid inference is achieved. In addition, the exclusion of observed components without an event from the analysis (eg, data from participants who are known to be alive, ie, no event in the mortality component, but whose culture results are missing) means that the derived endpoint may not be MCAR or missing at random (MAR), even when the components are MCAR.^6^

Maximum likelihood estimation has previously been considered for the assessment of treatment effect on a composite endpoint that is constructed from two or more partially observed components.^6,7,11^ This approach appears to work well when values of the components are MCAR or MAR. However, implementation in standard statistical software is limited, and incorporating baseline covariates in the analysis is not straightforward.

Multiple imputation (MI) has increasingly been used to handle missing data in trials, and is an alternative approach for the analysis of a composite endpoint with incomplete data in the components. MI is commonly performed assuming data are MAR. The application of MI in handling missing values in the components of a composite endpoint poses several practical questions, requiring further consideration.

- First, should MI be performed at the composite or component level?
- Second, when imputing at the composite level, should MI be performed on participants whose composite endpoint cannot be derived from their observed components, or on all participants whose data are missing in any components, regardless of whether their endpoint can be derived?
- Third, an essential condition for inference after MI to be valid is compatibility between the imputation and analysis models.^12–14^ If MI is to be used, how should the imputation model be specified so that the associations between the components, as well as between the composite endpoint and other variables in the substantive analysis model, are correctly reflected in the imputed data?

The aim of this paper is to examine a set of methods, readily available in common statistical software packages, for analyzing a binary composite endpoint with partially observed components. The remainder of this paper is organized as follows. In Section 2, we introduce and describe our motivating data set from the TOPPS trial.^15^ In Section 3, we consider the case of a simple composite endpoint with two components (one fully observed and one with missing values) and show algebraically that the endpoint derived from the observed component can be missing not at random (MNAR) even when the missing component is MCAR. Section 4 presents a simulation study which compares methods for handling missing data in the components for two types of composite endpoint. This shows that MI performed at the component level is generally preferable. If MI at the composite level is used, it should be performed on all participants whose data are missing in any components, and this approach only provides valid inference when the components are MCAR. Specifying the imputation model for MI at the component level requires careful consideration on the potential interactions between the components as well as with randomized treatment. A reanalysis of the TOPPS trial is presented in Section 5; and Section 6 concludes with a discussion.

## 2 Motivating Example: the Topps Trial

The trial of prophylactic platelets (TOPPS) was a randomized, open-label, noninferiority trial assessing whether a policy of not giving prophylactic platelet transfusions was as effective and safe as a policy of providing prophylaxis to prevent bleeding in patients with haematologic cancers.^15^ A total of 600 participants were recruited from 14 haematology centres in the UK and Australia between 2006 and 2011.

Eligible participants were 16 years or older who were undergoing, or were about to undergo, chemotherapy or stem-cell transplantation to treat a haematologic cancer, and who had, or were expected to have, thrombocytopenia. Participants were randomized in a 1:1 ratio to receive, or not to receive, prophylactic platelet transfusions. Bleeding assessment was conducted daily, and the primary outcome was the occurrence of at least one bleeding event in the 30 days after randomization (ie, a binary composite endpoint constructed from 30 binary indicators of whether the participant had a bleeding event on each day). The structure of this composite endpoint is the same as any other composite endpoint made up of “an event in any of the components”, and the missing bleeding assessments on some days means that this composite endpoint suffers from the same aforementioned issues.

Bleeding was experienced in 151 of 300 (50%) participants in the no-prophylaxis group, and 128 of 298 (43%) participants in the prophylaxis group. The trial reported an adjusted difference in proportions of 8.4%, 90% confidence interval (CI) 1.7% to 15.2%. Therefore, noninferiority of a no-prophylaxis strategy compared to a prophylaxis strategy for platelet transfusions was not declared based on a noninferiority margin of 15%.

For the primary analysis, MI was used to account for days with missing bleeding assessments. Briefly, the 30-day follow-up period was split into six time blocks of five days (ie, days 1 to 5, days 6 to 10, days 11 to 15, days 16 to 20, days 20 to 25, and days 26 to 30), and the number of bleeds occurring during each time block was counted. The number of bleeds in a time block was set to missing if three or more bleeding assessments were missing in that time block. For missing time blocks, the number of bleeds was then imputed from proportional odds models, conditional on the other time blocks and minimization variables, using the multivariate imputation by chained equations (MICE) approach.^16^

## 3 A Simple Composite Endpoint With Two Components

In this section, we explore the mathematical properties of the simplest binary composite endpoint with two binary components. We determine the missingness mechanism of the derived endpoint when one component is fully observed and the other component is MCAR. We also demonstrate the potential bias associated with an analysis of the derived endpoint compared with a CRA, and discuss model specification for MI.

Let *y* be a binary composite endpoint with two binary components *z*_1_ and *z*_2_; *y*, *z*_1_, *z*_2_ take values 0 or 1. We define a *simple* composite endpoint *y* as

$$y\;=\;\{\begin{array}{l} 1,\;\text{if}\;z_{1}=1\;\text{or}\;z_{2}=1\text{;}\;\\ 0,\;\text{if}\;z_{1}=0\;\text{and}\;z_{2}=0.\; \end{array}$$

Let *p_jk_* = P (*z*_1_ = *j* and *z*_2_ = *k*) ; *j, k* take values 0 or 1. Then P (*y* = 0) = *p*_00_ and P (*y* = 1) = *p*_01_ + *p*_10_ + *p*_11_. Further, suppose that *z*_1_ is fully observed for all participants, while *z*_2_ is missing for a subset of participants.

### 3.1 Missingness mechanism of the derived endpoint when one component is MCAR

When *z*_2_ is missing and *z*_1_ is observed, the composite endpoint *y* can be derived from the observed component *z*_1_ to take value 1 when *z*_1_ = 1, while *y* cannot be determined when *z*_1_ = 0. In other words, *y* is derivable from *z*_1_ = 1 regardless of the value of *z*_2_, whereas when *z*_1_ = 0 the value of *y* depends on what the missing value of *z*_2_ is, and in this case *z*_1_ alone does not provide sufficient information for *y* to be derived. This is because the composite *y* is defined as either *z*_1_ = 1 or *z*_2_ = 1.

We define *r*_*z*_2__ as the binary response indicator, taking values 1 when *z*_2_ is observed, and 0 otherwise. Let *r*_*y*_deriv__ denote the binary response indicator for the derived endpoint *y*_deriv_,

$$r_{y_{\text{deriv}}}\;=\;\{\begin{array}{l} 1,\;\text{if}\;r_{z_{2}}\;=\;1\;\text{or}\;\text{(}r_{z_{2}}\;=\;0\;\text{and}\;z_{1}\;=\;1\text{);}\;\\ 0,\;\text{if}\;r_{z_{2}}\;=\;0\;\text{and}\;z_{1}\;=\;0.\; \end{array}$$

Suppose *z*_2_ is MCAR with probability P (*r*_*z*_2__ = 1) = *α*, then

$$\begin{array}{l} \text{P(}r_{y_{\text{deriv}}}\;=\;0\text{)}\;\;=\;\;\text{P(}r_{z_{2}}\;=\;0\;\text{and}\;z_{1}\;=\;0\text{)}\;=\;(1-\alpha )(p_{00}+p_{01})\text{;}\\ \text{P(}r_{y_{\text{deriv}}}\;=\;1\text{)}\;\;=\;\;\text{P[}r_{z_{2}}\;=\;1\;\text{or}\;\text{(}r_{z_{2}}\;=\;0\;\text{and}\;z_{1}\;=\;1\text{)]}\;=\;\alpha +(1-\alpha )(p_{10}+p_{11})\text{.} \end{array}$$

The distribution of *y* among the subset of participants whose endpoint is considered missing is given by

(1) $$\begin{array}{l} \text{P(}y=1|r_{y_{\text{deriv}}}=0\text{)}=\frac{\text{P(}y=1\;\text{and}\;r_{y_{\text{deriv}}}=0\text{)}}{\text{P(}r_{y_{\text{deriv}}}=0\text{)}}\\ \;=\frac{\text{P(}r_{z_{2}}=0\;\text{and}\;z_{1}=0\;\text{and}\;z_{2}=1\text{)}}{\text{P(}r_{z_{2}}=0\;\text{and}\;z_{1}=0\text{)}}\\ \;=\frac{(1-\alpha )p_{01}}{\left(1-\alpha )(p_{00}+p_{01}\right)}\\ \;=\frac{p_{01}}{p_{00}+p_{01}}. \end{array}$$

Similarly, the distribution of *y* among participants with a derivable endpoint can be written as

(2) $$\begin{array}{l} \text{P(}y=1|r_{y_{\text{deriv}}}=1\text{)}=\frac{\text{P(}y=1\;\text{and}\;r_{y_{\text{deriv}}}=1\text{)}}{\text{P(}r_{y_{\text{deriv}}}=1\text{)}}\\ \;=\frac{\text{P(}y=1\;\text{and}\;r_{z_{2}}=1\text{)}+\text{P(}r_{z2}=0\;\text{and}\;z_{1}=1\text{)}}{\text{P(}r_{z_{2}}=1\text{)}+\text{P(}r_{z_{2}}=0\;\text{and}\;z_{1}=1\text{)}}\\ \;=\frac{\alpha (p_{10}+p_{01}+p_{11})+(1-\alpha )(p_{10}+p_{11})}{\alpha +(1-\alpha )(p_{10}+p_{11})}\\ \;=\frac{\alpha p_{01}+p_{10}+p_{11}}{\alpha +(1-\alpha )(p_{10}+p_{11})}. \end{array}$$

Since (1) ≠ (2) in general, *y*_deriv_ will likely be MNAR even when *z*_2_ is MCAR.

### 3.2 Bias in analysis of the derived endpoint versus complete records

#### 3.2.1 Analysis of the derived endpoint

In a randomized controlled trial, suppose we have a treatment variable *x* taking values 1 for treatment or 0 for control. Let *S_jk_* = P (*z*_1_ = *j* and *z*_2_ = *k* | *x* = 1) and *t*_jk_ = P (*z*_1_ = *j* and *z*_2_ = *k* | *x* = 0) ; *j, k* take values 0 or 1. When both components *z*_1_ and *z*_2_ are fully observed, the probability of *y* = 1 in the treatment and control arms is given by

(3) $$\text{P}(y=1|x=1)\;=\;s_{01}+s_{10}+s_{11}\;=\;1-s_{00};$$

(4) $$\text{P}(y=1|x=0)\;=\;t_{01}+t_{10}+t_{11}\;=\;1-t_{00}.$$

Suppose our effect measure of interest is an odds ratio (OR). From (3) and (4), the full-data OR for the treatment effect can be written as

(5) $${\text{OR}}_{\text{full}\;}\;=\;\frac{\text{P}(y=1|x=1)/\text{P}(y=0|x=1)}{\text{P}(y=1|x=0)/\text{P}(y=0|x=0)}\;=\;\frac{(s_{01}+s_{10}+s_{11})t_{00}}{(t_{01}+t_{10}+t_{11})s_{00}}\text{.}$$

With incomplete data, the distribution of the composite endpoint *y* among participants randomized to the treatment arm, whose endpoint can be derived from the values of *z*_1_, is

(6) $$\text{P(}y=1|r_{y_{\text{deriv}}}=1,x=1\text{)}\;=\;\frac{\alpha {\text{S}}_{01}+s_{10}+s_{11}}{\alpha +(1-\alpha )(s_{10}+s_{11})};$$

(7) $$\text{P(}y=0|r_{y_{\text{deriv}}}=1,x=1\text{)}\;=\;\frac{\alpha S_{00}}{\alpha +(1-\alpha )(s_{10}+s_{11})},$$

where *α* = P (*r*_*z*_2__ = 1). Similarly, the distribution of *y* among participants randomized to the control arm, whose endpoint is derivable from the values of *z*_1_, is

(8) $$\text{P(}y=1|r_{y_{\text{deriv}}}=1,x=0\text{)}\;=\;\frac{\alpha t_{01}+t_{10}+t_{11}}{\alpha +(1-\alpha )(t_{10}+t_{11})};$$

(9) $$\text{P(}y=0|r_{y_{\text{deriv}}}=1,x=0\text{)}\;=\;\frac{\alpha t_{00}}{\alpha +(1-\alpha )(t_{10}+t_{11})}.$$

From (6), (7), (8), (9), the OR for the treatment effect based on the derived endpoint is given by

(10) $${\text{OR}}_{\text{deriv}}\;=\;\frac{[\alpha S_{01}+s_{10}+s_{11}]t_{00}}{[\alpha t_{01}+t_{10}+t_{11}]s_{00}},$$

which, in general, is not equal to the OR given in (5) when the components are fully observed.

From (5) and (10), the ratio of OR_deriv_ to OR_full_ is given by

(11) $$\frac{{\text{OR}}_{\text{deriv}}}{{\text{OR}}_{\text{full}}}\;=\;\frac{\alpha S_{01}+s_{10}+s_{11}}{\alpha t_{01}+t_{10}+t_{11}}/\frac{S_{01}+s_{10}+s_{11}}{t_{01}+t_{10}+t_{11}}\;=\;\frac{1-(1-\alpha )\frac{s_{01}}{s_{01}+s_{10}+s_{11}}}{1-(1-\alpha )\frac{t_{01}}{t_{01}+t_{10}+t_{11}}}\;=\;\frac{1-(1-\alpha )\sigma }{1-(1-\alpha )\tau }.$$

From (11), the direction of bias in the OR due to missing data is determined by the relative sizes of *σ* and *τ*. OR_deriv_ will be inflated in analysis of the derived endpoint if *σ* < *τ*, and biased downwardly if *σ* > *τ*. An unbiased estimate of the OR is achieved when *σ* = *τ*, for example, when there is no effect of treatment on any of the components (ie, *S_jk_* = *t_jk_* for all *j*, *k*). The maximum magnitude of bias due to one component being MCAR will be to increase or decrease the OR by a factor of *α*.

To illustrate this, suppose P (*z*_1_ = 1 | *x* = 0) = P (*z*_2_ = 1 | *x* = 0) = 0.7, P (*z*_1_ = 1 | *x* = 1) = P (*z*_2_ = 1 | *x* = 1) = 0.2, and *z*_1_ ⫫ *z*_2_ | *x*. Then *σ* = 0.23, and *τ* = 0.44. If 70% of data in *z*_2_ are MCAR (ie, *α* = 0.3) then OR_deriv_ will be overestimated by 22%.

#### 3.2.2 Analysis of complete records

Suppose the analysis is performed on participants with observed data in both components, that is, *r*_*z*_2__ = 1. Then the distribution of the composite endpoint *y* among the complete records is the same as that when there are no missing data, as shown below.

(12) $$\begin{array}{l} \text{P(}y=1|r_{z_{2}}=1,x=1\text{)}=\frac{\text{P(}y=1\;\text{and}\;r_{z_{2}}=1|x=1\text{)}}{\text{P(}r_{z_{2}}=1|x=1\text{)}}\\ \;=\frac{\alpha (S_{01}+S_{10}+S_{11})}{\alpha }\\ \;=S_{01}+S_{10}+S_{11}\\ \;=\text{P}(y=1|x=1). \end{array}$$

It follows from (12) that, if the analysis discards participants with missing data in the incomplete component, the resulting estimated treatment effect will be unbiased.

### 3.3 MI of the incomplete component

When data in *z*_2_ are missing (with *z*_1_ fully observed), MI can be performed either at the composite level, that is, *y* is imputed directly, or at the component level, that is, *z*_2_ is imputed first and then *y* is passively imputed from *z*_1_ and *z*_2_.

For MI at the composite level, *y* can be imputed whenever *z*_2_ is missing, regardless of the values of *z*_1_ (MI-CRA). Alternatively, *y* can be derived from the values of *z*_1_ first before the remaining missing (nonderivable) values in *y* are imputed (MI-Deriv).

Suppose the substantive analysis model is a logistic regression model for the composite endpoint *y*, conditional on randomized treatment *x*. Then *x* needs to be included in the imputation model for *y* to ensure compatibility between the imputation and analysis models.^14^

Specification of the imputation model at the component level, that is, when *z*_2_ is imputed, is more complex. Both the fully observed component *z*_1_ and randomized treatment *x* should be included in the imputation model for *z*_2_. However, the imputation model for *z*_2_ can be specified in several ways, by:

- including *x* and *z*_1_ as main effects (MIC-main);
- including *z*_1_ as main effect and stratifying the imputation by *x*, so that the association between *z*_2_ and *z*_1_ varies by *x* (MIC-*x*); or
- stratifying the imputation by both *x* and *z*_1_, so that the distribution of *z*_2_ differs across strata defined by values of *x* and *z*_1_ (MIC-*x*-*z*_1_).

The correct specification of the imputation model depends on the true associations between *z*_1_, *z*_2_, and *x*. Note that in this example the last imputation model will never be mis-specified but, as usual, there is a balance between the ability to be unbiased for any given data generating mechanism, and the practical chance that the imputation model will not converge for a given sample size and data set. The simulation study presented in the next section explores these MI approaches in more detail.

## 4 Simulation Study

### 4.1 Design

#### 4.1.1 Aims

We conducted a simulation study to explore the statistical properties of a set of methods for handling missing values in the components of a composite endpoint (described in Section 3.3), as well as to support our analytic results in Section 3.

#### 4.1.2 Data generating mechanism

We considered the case of a randomized controlled trial in which participants are randomized by simple randomization with equal probability to either the treatment or control arm (denoted by *x*, taking values 1 or 0, respectively). For each participant, a binary composite endpoint *y* is constructed from three binary components *z*_1_, *z*_2_, *z*_3_; *y* and the *z*s take values 0 or 1. Two examples of how a composite endpoint may be constructed from three components, which we refer to as *simple* and *complex* composite endpoints, were considered, where

$$y_{\text{simple}}=\{\begin{array}{l} 1,\;\text{if}\;z_{1}=1\;\text{or}\;z_{2}=1\;\text{or}\;z_{3}=1\text{;}\\ 0,\;\text{if}\;z_{1}=0\;\text{and}\;z_{2}=0\;\text{and}\;z_{3}=0\text{;} \end{array}$$

and

$$y_{\text{complex}}\;=\;\{\begin{array}{l} 1,\;\text{if}\;z_{1}=1\;\text{and}\;\text{(}z_{2}=1\;\text{or}\;z_{3}=1\text{);}\\ 0,\;\text{otherwise.} \end{array}$$

When data in the components are completely observed, there are eight combinations of these components from which the values of *y* are determined (Table 1). In this simulation study, we first generated data in the components and then used them to construct the composite endpoint. To control the associations between the components, we defined a saturated log-linear model for the count of each combination *c*,

(13) $$\log(\mu_{c})\;=\;\mu_{0}+{\text{LP}}_{c},\;c=1,\;\ldots \;,8,$$

where LP_*c*_ is the linear predictor and *μ*_0_ is the intercept term included in the model for the counts to sum to the total number of participants. LP_*c*_ can be written in terms of the components as

(14) $${\text{LP}}_{c}\;=\;\lambda_{1}z_{1}+\lambda_{2}z_{2}+\lambda_{3}z_{3}+\lambda_{12}z_{1}z_{2}+\lambda_{23}z_{2}z_{3}+\lambda_{13}z_{1}z_{3}+\lambda_{123}z_{1}z_{2}z_{3},$$

where *λ*_12_, *λ*_23_, *λ*_13_ correspond to the pairwise log ORs between any two components when the remaining component takes value 0, and *λ*_123_ represents the interaction between any two components in a logistic regression model with the remaining component as the dependent variable.

Then the probability of each combination is given by

(15) $$p_{c}\;=\;\frac{\exp({\text{LP}}_{c})}{\sum_{c=1}^{8}\exp({\text{LP}}_{c})}.$$

The expressions for the linear predictor corresponding to the eight combinations are presented in Table 1. It follows that the probability of meeting the composite endpoint is

(16) $$\text{P(}y_{\text{simple}}=1\text{)}\;=\;\sum_{c=2}^{8}p_{c};$$

(17) $$\text{P(}y_{\text{complex}}=1\text{)}\;=\;\sum_{c=6}^{8}p_{c}.$$

We considered three cases for the associations between the components and randomized treatment, where

- *λ*_123_ = 0 in both treatment and control arms;
- *λ*_123_ = 0 in the treatment arm but ≠ 0 in the control arm;
- *λ*_123_ ≠ 0 in both arms, with a different value in each arm.

These cases were considered in order to assess the validity of MI at the component level under potential mis-specification of the imputation model.

In addition, we assumed that data in *z*_1_ are fully observed, while *z*_2_ and *z*_3_ contain missing values generated under three missingness mechanisms (described later in this section).

The procedure for generating complete data was as follows.

- Generate *N*_sample_ = 2 000 complete values of a binary treatment variable *x* taking values 0 or 1 from the model
  $$x\;∼\;\text{Bernoulli(}p_{x}=0.5\text{)},$$
  reflecting simple randomization, with the sample size chosen to reduce small-sample bias associated with logistic regression;
- Separately for each treatment arm, generate a categorical variable *c* which takes values 1 to 8 from (15), with values of *λ*s selected to give a control arm event rate of 0.57 and event rate in the intervention arm of 0.84 (Supplementary Table S1);
- Generate three components from *c* with values corresponding to those in Table 1, that is,
  – *z*_1_ = 1 if *c* > 4; and 0 otherwise;
  – *z*_2_ = 1 if *c* = 3, 4, 7, 8; and 0 otherwise;
  – and *z*_3_ = 1 if *c* = 2, 4, 6, 8; and 0 otherwise;

Finally, generate a binary composite endpoint *y* taking values 0 or 1 from the three components *z*s (Table 1). With the values of *λ*s given in Supplementary Table S1, the effect of treatment *x* on the composite endpoint *y* is given by

(18) $$\text{logit}\;[\text{P}(y=1|x)]\;=\;\beta_{0}+\beta_{x}x,$$

where, for both simple and composite endpoints, *β*_0_ and *β_x_* are equal to 0.3 and 1.35, respectively.

Missing data were then introduced as follows.

- Generate binary indicators of response *r_l_* of *z_l_* from the following model
  (19) $$\text{logit}\;[\text{P(}r_{z_{1}}=1|z_{1},x\text{)}]\;=\;\alpha_{0}+\alpha_{x}x+\alpha_{z_{1}}z_{1}+\alpha_{xz_{1}}xz_{1},\;l\;=\;2,3,$$
  where
  (i) *α*_0_ = 0.7, *α_x_* = *α*_*z*_1__ = *α*_*xz*_1__ = 0, corresponding to a MCAR mechanism;
  (ii) *α*_0_ = 1.05, *α_x_* = -0.75, *α*_*z*_1__ = 0.25, *α*_*xz*_1__ = 0, corresponding to the first MAR mechanism (MAR1); and
  (iii) *α*_0_ = 1.05, *α_x_* = -0.75, *α*_*z*_1__ = 0.25, *α*_*xz*_1__ = 0.25, corresponding to the second MAR mechanism (MAR2).
  Under each of these three missingness mechanisms, the probability of observing each component is around 0.7, and the probability of observing all components is around 0.49;
- For *l* = 2, 3, set *z_l_* to missing if *r_z_l__* = 0.

**Figure 1:**
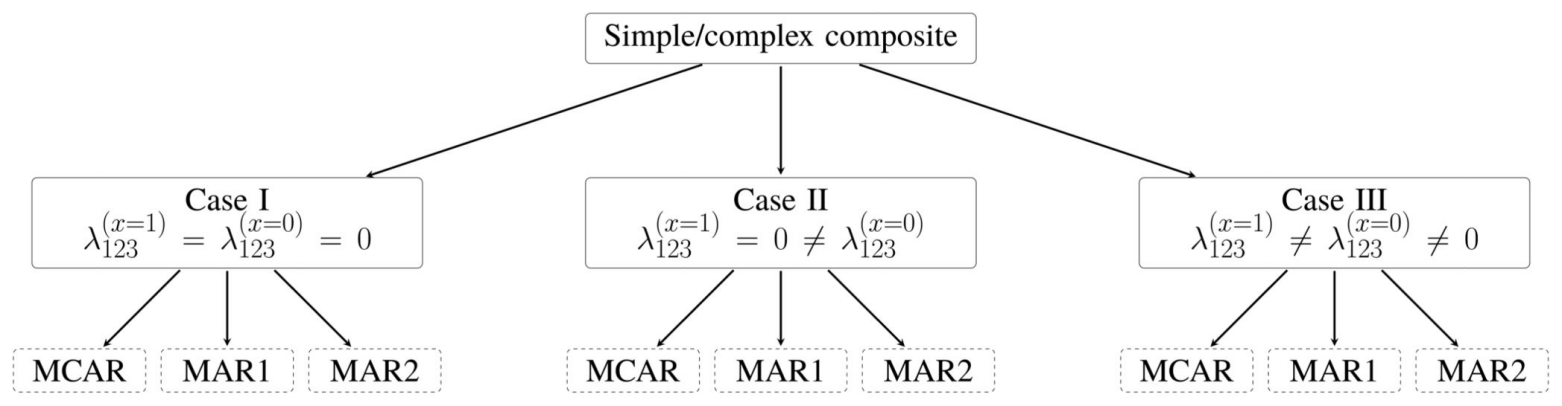
Simulation study: simulation scenarios for simple and complex composite endpoints; each combination in the dashed boxes was repeated independently *N*_rep_ = 2 000 times. *x*, randomized treatment; *λ*_123_, three-way interaction between the components in the log-linear model

These steps were repeated *N*_rep_ = 2 000 times under each of the nine scenarios of cases I to III and missingness mechanisms MCAR, MAR1, MAR2, for simple and complex composite endpoints separately (Figure 1). The number of simulation repetitions was chosen to produce a Monte Carlo error of 0.5% on a coverage of 95%.

#### 4.1.3 Estimands

The estimand is the log odds ratio *β_x_* for the treatment effect, whose true value is 1.35.

#### 4.1.4 Methods of analysis

We compared the following methods for handling missing values in *z*_2_ and *z*_3_ (Table 2).

- *CRA*: perform a complete record analysis, excluding from analysis participants with missing values in either component;
- *Deriv*: derive *y* from the observed components when possible, exclude from analysis participants whose *y* cannot be derived and is considered missing;
- *MI-CRA* (MI of the composite endpoint): perform MI of *y* whenever a component is missing, regardless of whether *y* is derivable from the observed components. The imputation model for the composite endpoint is conditional on the randomized treatment *x*;
- *MI-Deriv* (MI of the composite endpoint): derive *y* from the observed components when possible, perform MI of *y* for the remaining missing values. The imputation model for the composite endpoint is conditional on the randomized treatment *x*;
- *MIC-main* (MI of the components): perform MI of *z*_2_ and *z*_3_ using MICE; the conditional model for each component includes the randomized treatment *x*, the fully observed component *z*_1_, and the other incomplete component as main effects; *y* is passively imputed from the observed and imputed components.
- *MIC-x* (MI of the components): perform MI of *z*_2_ and *z*_3_ using MICE; the conditional model for each component includes the fully observed component *z*_1_ and the other incomplete component as main effects, and imputation is stratified by randomized treatment *x*; *y* is passively imputed from the observed and imputed components.
- *MIC-x-z*_1_ (MI of the components): perform MI of *z*_2_ and *z*_3_ using MICE; the conditional model for each component includes the other incomplete component as main effect, and imputation is stratified by the fully observed component *z*_1_ and randomized treatment *x*; *y* is passively imputed from the observed and imputed components.

For all MI methods, results from the imputed data sets were pooled using Rubin’s rules.^17^ From the chosen values of *λ*s (Supplementary Table S1) the imputation model at the component level that is compatible with the substantive analysis model for case I is MIC-*x*; *z*_2_ was imputed from the following conditional model

$$\text{logit}\;[\text{P(}z_{2}=1|z_{1},z_{3},x\text{)}]\;=\;\gamma_{0}+\gamma_{1}z_{1}+\gamma_{3}z_{3}+\gamma_{x}x+\gamma_{1x}z_{1}x+\gamma_{3x}z_{3}x,$$

and similarly for *z*_3_, with *z*_2_ as predictor.

For cases II and III, the compatible MI strategy at the component level is MIC-*x*-*z*_1_. The following conditional model was used to impute *z*_2_ (and similarly for *z*_3_, with *z*_2_ as predictor)

$$\text{logit}\;[\text{P(}z_{2}=1|z_{1},z_{3},x\text{)}]\;=\;\gamma_{0}+\gamma_{1}z_{1}+\gamma_{3}z_{3}+\gamma_{x}x+\gamma_{13}z_{1}z_{3}+\gamma_{1x}z_{1}x+\gamma_{3x}z_{3}x+\gamma_{13x}z_{1}z_{3}x.$$

#### 4.1.5 Performance measures

Bias, efficiency of ${\hat{\beta }}_{x}$ (in terms of the empirical and average model standard errors), and coverage of 95% CIs were calculated for each of the nine simulation scenarios,^18,19^ with analyses of full data (ie, before any values in *z*_2_ and *z*_3_ are set to missing) provided for comparison. These performance measures are defined as follows.

- Bias: $\text{E}\;[\hat{\beta }]\;-\;\beta ;$
- Empirical standard error: $\sqrt{\text{Var}\;(\hat{\beta })};$
- Average model standard error: $\sqrt{\text{E}[\hat{Var}(\hat{\beta })]};$
- Coverage: $\text{P}\;\text{(}{\hat{\beta }}_{\text{low}}\leq \beta \leq {\hat{\beta }}_{\text{upp}}\text{).}$

All simulations were performed in Stata/MP 15.1^20^ (the code is available at https://github.com/mytrapham/misscomposite); mi impute logit and mi impute chained were used for creating the imputations at the composite level and component level, respectively, and mi estimate for fitting the analysis model to the imputed data sets and pooling the results. Simulation results were analyzed using the community-contributed command simsum.^19^

### 4.2 Results

#### 4.2.1 Simple composite endpoint

**Figure 2:**
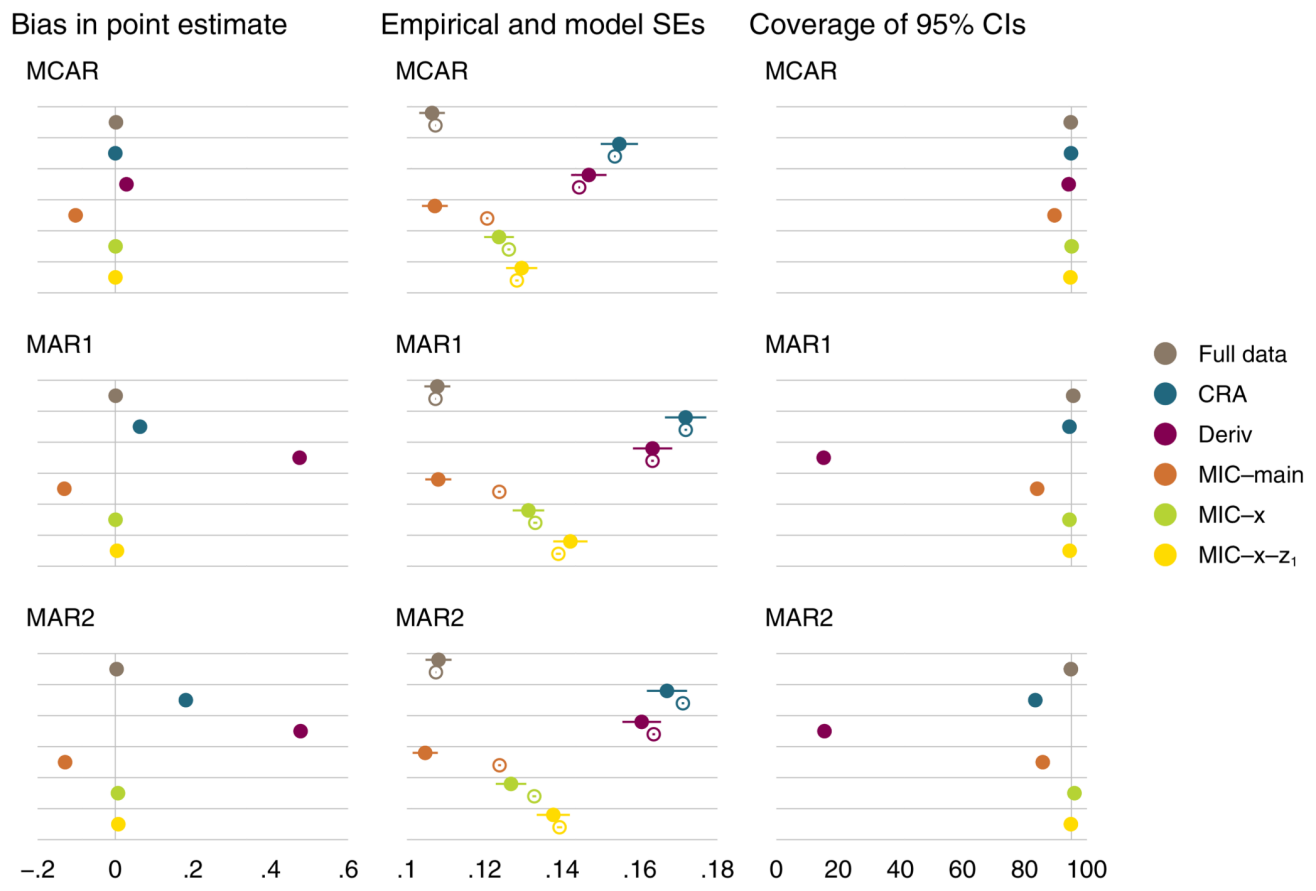
Simple composite endpoint, case I: performance measures for ${\hat{\beta }}_{x}$ under different missingness mechanisms of the components; *β_x_* = 1.35. Error bars, ±1.96× Monte Carlo errors; filled and hollow points, empirical and average model standard errors, respectively; vertical lines at 0 and 95 for bias and coverage, respectively [Colour figure can be viewed at wileyonlinelibrary.com]

**Figure 3:**
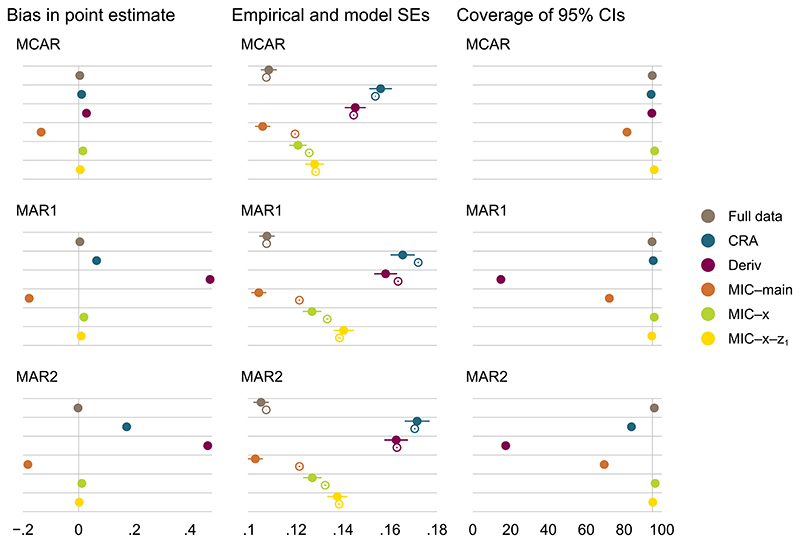
Simple composite endpoint, case II: performance measures for ${\hat{\beta }}_{x}$ under different missingness mechanisms of the components; *β_x_* = 1.35. Error bars, ±1.96× Monte Carlo errors; filled and hollow points, empirical and average model standard errors, respectively; vertical lines at 0 and 95 for bias and coverage, respectively [Colour figure can be viewed at wileyonlinelibrary.com]

**Figure 4:**
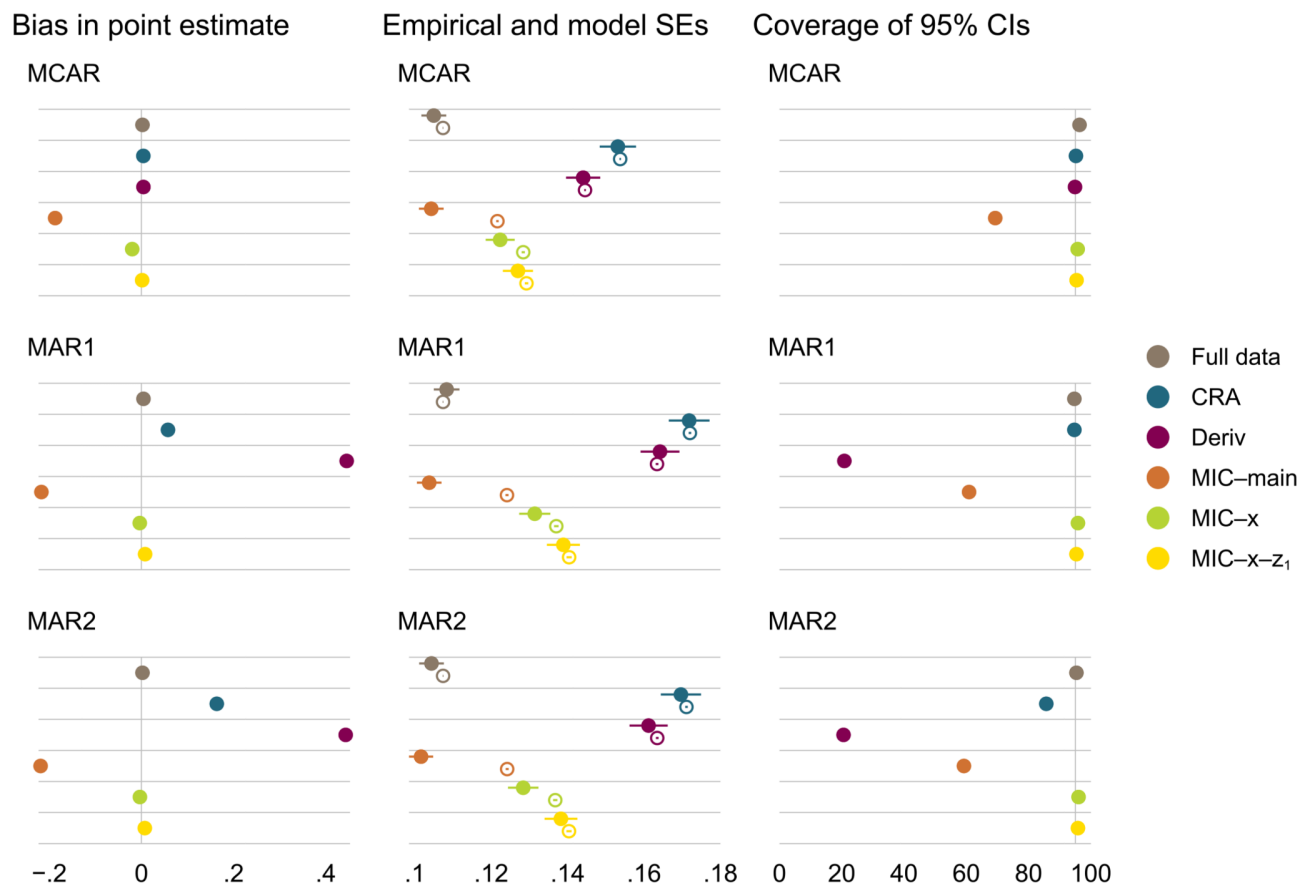
Simple composite endpoint, case III: performance measures for ${\hat{\beta }}_{x}$ under different missingness mechanisms of the components; *β_x_* = 1.35. Error bars, ±1.96× Monte Carlo errors; filled and hollow points, empirical and average model standard errors, respectively; vertical lines at 0 and 95 for bias and coverage, respectively [Colour figure can be viewed at wileyonlinelibrary.com]

Simulation results for a simple composite endpoint are summarized graphically in Figures 2,3, and 4 for ${\hat{\beta }}_{x}$ (ie, our main estimand); results for ${\hat{\beta }}_{0}$ are presented in Supplementary Figures S1 to S3 for reference.

Analysis of full data is unbiased with the smallest standard errors and coverage at the nominal 95% level. MI-CRA and MI-Deriv produce very similar results to CRA and analysis of the derived endpoint, respectively; hence, their results are not presented. This is because for MI at the composite level the imputation and analysis models are identical, and MI results only reflect additional Monte Carlo errors.

$$Case\;I\text{:}\;\;\lambda_{123}^{\left(x=1\right)}\;=\;\lambda_{123}^{\left(x=0\right)}\;=\;0$$

CRA is unbiased when the components *z*_2_ and *z*_3_ are MCAR. Under the posited MAR mechanisms where the components are missing conditional on both *z*_1_ (fully observed) and randomized treatment *x*, the composite endpoint *y* is thus MNAR conditional on its values, in which case CRA provides biased estimates of *β*s as the theory suggests. If we instead consider a MAR mechanism where *z*_2_ and *z*_3_ are missing conditional only on randomized treatment *x*, then CRA will be unbiased.

Analysis of the derived endpoint is biased across all missingness mechanisms considered, consistent with the analytic results (Section 3). Bias is severe in both parameter estimates, apart from the log odds ratio ${\hat{\beta }}_{x}$ under MCAR, where bias is minimal. This might be due to bias in the treatment and control log odds being cancelled out when used to calculate the log OR.

MI at the component level with randomized treatment *x* and fully observed component *z*_1_ as main effects in the conditional imputation models (MIC–main) is biased, as the two-way interactions between the components and randomized treatment are omitted in the imputation model. By contrast, MI at the component level with *z*_1_ as main effect and stratified by *x* (MIC-*x*) is unbiased, as it is the correct model in this scenario. Since MI at the component level stratified by both *x* and *z*_1_ (MIC-*x*-*z*_1_) is a more general model of MIC-*x*, it is also correct and unbiased. For scenarios where both MI at the component level and CRA are valid methods, MI is more efficient than CRA.

$$Case\;II\text{:}\;\;\lambda_{123}^{\left(x=1\right)}\;=\;0\;\neq \;\lambda_{123}^{\left(x=0\right)}\text{;}\;\;case\;III\text{:}\;\lambda_{123}^{\left(x=1\right)}\;\neq \;\lambda_{123}^{\left(x=0\right)}\;\neq \;0$$

Results under cases II and III are similar to those seen under case I. While MIC-*x*-*z*_1_, which accounts for the three-way interaction between the components and randomized treatment in the conditional imputation models, is the only correct approach in these cases, bias in MIC-*x* appears to be minimal for both parameter estimates across the missingnessmechanisms. Bias in MIC-*x* may be more apparent with other choices of parameter values in the data generating mechanism.

#### 4.2.2 Complex composite endpoint

Simulation results for the complex composite endpoint are summarized graphically in Supplementary Figures S4 to S9 (for both ${\hat{\beta }}_{x}$ and ${\hat{\beta }}_{0});$ they are similar to results for the simple composite endpoint. MI at the component level occasionally suffered from perfect prediction (often termed *separation*) when imputation was stratified by randomized treatment *x* and fully observed component *z*_1_ (MIC-*x*-*z*_1_); however, all occurrences of perfect prediction were overcome when augmentation was used in MI (via the specification of option augment in mi impute, Supplementary Table S2).^21^ This approach involves “augmenting” the data set by adding a few extra observations with small weights to the data during estimation of model parameters in a way that overcomes perfect prediction.^21^

## 5 Reanalysis of the Topps Trial

### 5.1 Methods of analysis

The composite endpoint in TOPPS was a simple composite endpoint constructed from 30 daily bleeding assessments, with an outcome event occurring if the participant experienced at least one bleeding event. We anticipated perfect prediction to be an issue when performing MI at the component level with 30 components. Thus, following what had been done in the original TOPPS analysis, we split the 30-day follow-up period into six time blocks, each of five days.

We considered two approaches for defining the completeness of these six blocks; the latter was how block-level completeness had been defined in the original TOPPS analysis.

- Approach 1: each block was set to missing if bleeding status was missing for any of the five days;
- Approach 2: each block was set to missing if bleeding status was missing for at least three of the five days.

Our main focus was missing data at block level. Since most of the missing data were at block level, we used relatively ad hoc methods to handle missing data within blocks. We handled missing data within blocks by a CRA approach (approach 1); as a sensitivity analysis we also derived the bleeding status for the blocks (approach 2). For blocks that were not set to missing (according to approaches 1 and 2), each block took value 1 if there was at least one bleeding event during the five days (ie, an initial block-wise derivation step in approach 2). These six blocks were then used to construct the composite endpoint, which took value 1 if any block took value 1, and 0 if all blocks took values 0.

In this reanalysis, we compared the following methods for handling missing values in the six time blocks: (i) CRA; (ii) Deriv; (iii) MI-CRA; (iv) MI–Deriv; (v) MIC-main; and (vi) MIC-trt. For MIC-main, we performed MI of the blocks using MICE, where the conditional imputation model for each block included the randomized treatment and other incomplete blocks as main effects. For MIC-trt, blocks were imputed using MICE; the conditional model for each block included other incomplete blocks as main effects, and imputation was stratified by the randomized treatment. Since none of the blocks were fully observed, MI at the component level stratified by the randomized treatment and fully observed component(s) (ie, a version of MIC-*x*-*z*_1_ in Section 4) was not relevant here. All MI methods were performed using 50 imputations and 20 burn-in cycles.

Initially, MIC-trt was performed using Stata’s mi impute chained (MIC-trt 1). However, perfect prediction led to nonconvergence in one of the imputations which caused MI to break down, and specifying the augment option did not help overcome this. We therefore considered two alternatives: (i) use the community-contributed command ice^22^ (MIC-trt 2); and (ii) use mi impute chained, but imputing each block conditional on two adjacent blocks instead of all other blocks (MIC-trt 3). These two alternatives successfully imputed missing values in the incomplete time blocks.

As in the original TOPPS analysis, our substantive analysis model was a generalized linear model for the composite endpoint (constructed from six time blocks) on randomized treatment, with an identity link and binomial family. For simplicity, minimization variables used in the original TOPPS analysis were not included in our substantive analysis and imputation models. Our estimand was the difference in proportions of participants who had bleeding events between the two treatment arms (no-prophylaxis versus prophylaxis platelet transfusion).

### 5.2 Results

Of the 600 participants, the majority did not have any missing bleeding assessments in any of the six time blocks (Supplementary Table S3). When treating a block as missing if any bleeding assessment was missing (ie, approach 1), 462 (77%) participants had complete data in all six time blocks, and 9 (2%) had missing data in all six time blocks. The remaining 129 (21%) participants had between one and five incomplete time blocks. The 462 (77%) participants with complete data were included in the CRA, while Deriv used data from 518 (86%) participants, those with complete data for all blocks, or at least one nonmissing block in which a bleeding event was recorded.

In approach 2 (ie, treating a block as missing if at least three of the five bleeding assessments were missing), 553 (92%) participants had complete data in all six time blocks, and 5 (1%) had missing data in all blocks. The rest of the participants (42; 7%) had between one and five incomplete time blocks. CRA included 553 (92%) participants with complete data; Deriv was performed on 576 (96%) participants whose endpoint was derivable from the observed time blocks.

**Figure 5:**
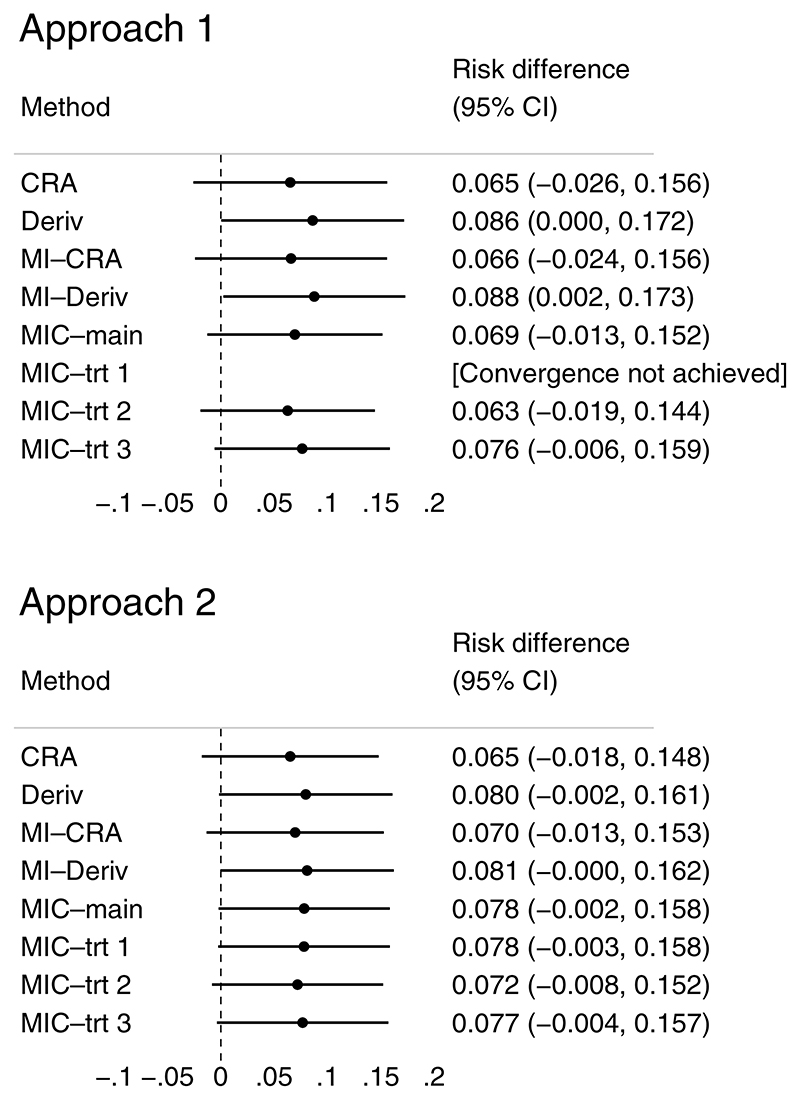
TOPPS reanalysis: difference in proportions of participants who had bleeding events between the two treatment arms under different methods for handling missing bleeding events. MIC-trt 1, MI performed by mi impute chained, imputation of each block is conditional on all other blocks and stratified by randomized treatment; MIC-trt 2, MI performed by ice, imputation of each block is conditional on all other blocks and stratified by randomized treatment; MIC-trt 3, MI performed by mi impute chained, imputation of each block is conditional on two adjacent blocks and stratified by randomized treatment

Figure 5 presents the difference in proportions of participants who had bleeding events between the two treatment arms under different methods for handling missing bleeding events. The estimated proportions by randomized treatment are given in Supplementary Table S4. For MI methods, Monte Carlo errors for the estimated differences are less than 10% of the corresponding estimated standard errors with 50 imputations.

Apart from Deriv and MI-Deriv, results are generally comparable across methods, which are also similar to the original TOPPS analysis result (risk difference 0.084, 90% CI 0.017 to 0.152). MI-CRA and MI-Deriv are similar to CRA and Deriv, respectively, as seen in Section 4. Deriv and MI-Deriv produce the largest estimated differences in both approaches, and are the only methods that are statistically significant under a superiority design (in approach 1). These results are in line with our analytic and simulation results for Deriv and MI-Deriv. MI methods performed at the component level produce estimates that are more efficient than CRA, with narrower CIs.

## 6 Discussion

When analyzing a binary composite endpoint with nonsimultaneously missing data in the components, a strategy frequently used in practice is to derive the endpoint from the observed components when possible and discard data from participants whose endpoint cannot be derived. By exploring the missingness mechanism of the derived endpoint both mathematically and by simulation, we showed that even when the components are MCAR, the composite endpoint derived from the observed components can be MNAR. As a result, an analysis of the derived endpoint will be biased. Omitting from analysis participants with missing data in the components (ie, a CRA) can reduce efficiency when the components are MCAR, and lead to bias when the components are MAR.

Our simulation study compared a set of methods, readily available in common statistical software packages, for handling missing values in the components of a binary composite endpoint. MI is a natural approach, and performing MI at the component level is generally preferable. Imputing the incomplete components when they are MCAR can improve efficiency compared with a CRA or MI at the composite level (MI-CRA). Under complex MAR mechanisms of the components, valid inference can be achieved with MI at the component level. By defining a model for the relations between the components in the data generating mechanism of our simulation design, we demonstrated that the choice of imputation model for the incomplete components might not be straightforward. The correct choice depends on the interactions between the components and also with randomized treatment. In the scenarios examined in our simulation study, MICE with conditional imputation models for the incomplete components, stratified by the randomized treatment and fully observed component (ie, allowing for the distribution of the incomplete components to differ across strata defined by values of the randomized treatment and fully observed component), is generally the preferred approach to other specifications of MI under consideration.

For nonmonotone patterns of missing data, the two standard model-based MI approaches are MICE^16^ and joint model imputation;^13^ theoretical equivalence of these two approaches in certain settings has been explored previously.^23,24^ While MICE involves specifying a series of conditional imputation models for the incomplete variables, joint model imputation is commonly based on the specification of a multivariate normal distribution for the incomplete variables. Here our MI results were obtained using MICE for the incomplete binary components, but alternatively these components could be imputed using the joint model imputation approach. When joint model imputation is performed for incomplete binary variables, one approach is to treat them as continuous in the imputation model, which means the imputed variables can take values other than 0/1. An additional rounding step could be used, but some approaches to rounding have been shown to yield bias in certain settings.^25,26^ Thus, joint model imputation might not be appropriate for the incomplete binary components considered in our simulation study and the TOPPS trial. In addition, an advantage of MICE is that the method is more flexible in handling missing values in several variables of different types. Here we considered the setting where all incomplete variables to be imputed are binary components of the composite endpoint, but in practice we might also need to impute other incomplete variables which are, for example, continuous, alongside the binary components.

In this article, we explored a binary composite endpoint constructed from two or more binary components. Unlike the setting investigated by O’Keeffe et al^9^ (described in Section 1), we examined the scenario where the components are not always missing (MCAR/MAR) simultaneously, and thus the composite endpoint can be derived from the components depending on their observed values. This difference in the missingness pattern has implications for whether imputation should be performed at the composite or component level, as has been shown in our simulation study.

Although we did not consider a composite endpoint that is the time to the first of two or more events, whichever occurs first (as described in Section 1), our finding about potential bias associated with deriving the endpoint from observed components can still apply to this type of composite endpoint. MI at the component level is also possible, although it is potentially more complex since the imputation needs to be performed for both the time to event and event indicator.

In the reanalysis of the TOPPS trial, we chose to split the 30-day period into six time blocks of five days as had been done in the original analysis of the trial. Other ways of splitting the follow-up period into time blocks could also be considered. For example, in the most extreme case, we could even consider splitting this period into 30 blocks of one day; however, given the size of the TOPPS data set, performing MI of 30 components while allowing for the imputation to be stratified by randomized treatment would likely result in nonconvergence. In fact, even with six blocks of five days, convergence was not achieved for one of the methods considered (MIC-trt 1) under approach 1 used for defining the completeness of these six blocks (Figure 5, Section 5.2). The choice of block size requires practical consideration on the ability to be unbiased for any given data generating mechanism, while accounting for potential issues related to nonconvergence of the imputation model for a given sample size and data set.

MI allows for the inclusion of auxiliary variables in the imputation model. Good candidates for auxiliary variables are those that are predictive of both the missing values and the probability of data being missing.^27^ Including these auxiliary variables in the imputation model will improve the plausibility of the MAR assumption and reduce bias. Auxiliary variables that are only predictive of the missing values can help to reduce the standard errors of estimates in the analysis model.^27^ In the reanalysis of the TOPPS trial, the inclusion of such auxiliary variables (if available) could improve the performance of MI, although whether additional interaction terms need to be specified in the conditional imputation models requires further exploration.

The reanalysis of the TOPPS trial suggested that results were relatively robust to the choice of method for handling missing values in the components (ie, six blocks of five daily bleeding assessments) of the composite endpoint. However, CRA produced the widest CI and represents a potential waste of resources. Compared with other methods under comparison, Deriv and MI-Deriv produced the largest estimated differences. They were also the only methods that changed the statistical significance of the results under a superiority design, which might be explained by the bias demonstrated in our analytic and simulation results. This bias can also negatively impact the results of a noninferiority analysis. In practice, bias associated with using the derived endpoint can potentially change the conclusion of the trial.

Our results highlighted the need to give careful consideration to the choice of method for handling missing data in the components when analyzing a composite endpoint. Although superficially attractive, an analysis of the derived endpoint should generally be avoided or used with extreme caution. Despite the risk of imputation model mis-specification, we showed that MI at the component level is the preferred approach in this study setting.

## Supplementary Material

Supporting Information

## Figures and Tables

**Table 1:** Simulation study: all possible combinations of the components for constructing the simple and complex composite endpoints, and associated linear predictors in the log-linear model for the combinations of components

Combination *c*	*Z*_1_	*Z*_2_	*Z*_3_	*y*_simple_	*y*_complex_	Linear predictor LP_*c*_ for log (*μ_c_*)
1	0	0	0	0	0	0
2	0	0	1	1	0	*λ*_3_
3	0	1	0	1	0	*λ*_2_
4	0	1	1	1	0	*λ*_2_ + *λ*_3_ + *λ*_23_
5	1	0	0	1	0	*λ*_1_
6	1	0	1	1	1	*λ*_1_ + *λ*_3_ + *λ*_13_
7	1	1	0	1	1	*λ*_1_ + *λ*_2_ + *λ*_12_
8	1	1	1	1	1	*λ*_1_ + *λ*_2_ + *λ*_3_ + *λ*_12_ + *λ*_23_ + *λ*_13_ + *λ*_123_

**Table 2:** Simulation study: methods for handling missing values in partially observed components *z*_2_ and *z*_3_. y, composite endpoint; *x*, randomized treatment; *z*_1_, fully observed component

Method	Variable(s) imputed	Imputation model predictors
CRA		
Deriv		
^a^MI-CRA	*y*_CRA_	*x*
^a^MI-Deriv	*y*_deriv_	*x*
^b^MIC-main	*z*_2_, *z*_3_	*z*_1_, *z*_2_ or *z*_3_, *x*
^b^MIC-*x*	*z*_2_, *z*_3_	*z*_1_, *z*_2_ or *z*_3_; stratified by *x*
^b^MIC-*x*-*z*_1_	*z*_2_, *z*_3_	*z_2_* or *z*_3_; stratified by *z*_1_ and *x*

a Univariate MI using logistic regression.

b MICE using logistic regression for conditional models.

## Data Availability

The code and data used in the simulation study (Section 4) are available at https://github.com/mytrapham/misscomposite. The data used in the TOPPS reanalysis (Section 5) are available from the corresponding author of the original TOPPS publication (SJS) upon request; the code is available at https://github.com/mytrapham/misscomposite.
