# Supplementary material for: A comparison of methods for analyzing a binary composite endpoint with partially observed components in randomized controlled trials
Source: Stat Med. Author manuscript; Available in PMC 2023 Jun 15. (PMC7614656; doi:10.1002/sim.9203)
Supplement: Supporting Information [file EMS176925-supplement-Supporting_Information.pdf]

Supplementary materials

A comparison of methods for analyzing a binary composite endpoint with partially observed components in randomized controlled trials

Tra My Pham[1], Ian R White[1], Brennan C Kahan [1], Tim P Morris[1], Simon J Stanworth[2], Gordon Forbes[3]

[1]MRC Clinical Trials Unit at UCL, Institute of Clinical Trials and Methodology, London, UK

[2]NHS Blood & Transplant, Oxford University Hospitals and the University of Oxford, Oxford, UK

[3]Biostatistics & Health Informatics Department, King's College London, London, UK

S1 Simulation study

S1.1 Design

Table S1 presents values of the parameters in the log-linear model used to generate data in the components of a simple or complex composite endpoint. These parameters were selected to give a control arm event rate of 0.57 and event rate in the intervention arm of 0.84. We set the values of the two-way and three-way parameters, and used these together with the predefined log odds and log odds ratio (0.3 and 1.35, respectively) to derive values of the main effect parameters. For simplicity, main effect parameters were constrained to take the same values, λ_c and λ_t, for the control arm and treatment arm, respectively. The procedure for deriving parameter values corresponding to the simple and complex composite endpoints is presented in Sections S1.1.1 and S1.1.2.

S1.1.1 Simple composite endpoint

$$p\,(y=0) = \frac{\exp\,(0)}{\sum_{\text{LP}}},$$
$$\rightarrow \sum_{\text{LP}} = \frac{1}{p\,(y=0)}.$$

$$\begin{aligned} p\,(y=1) = {} & \frac{\exp\,(\lambda_1)}{\sum_{\text{LP}}} + \frac{\exp\,(\lambda_2)}{\sum_{\text{LP}}} + \frac{\exp\,(\lambda_3)}{\sum_{\text{LP}}} \\ & + \frac{\exp\,(\lambda_1+\lambda_2+\lambda_{12})}{\sum_{\text{LP}}} + \frac{\exp\,(\lambda_1+\lambda_3+\lambda_{13})}{\sum_{\text{LP}}} + \frac{\exp\,(\lambda_2+\lambda_3+\lambda_{23})}{\sum_{\text{LP}}} \\ & + \frac{\exp\,(\lambda_1+\lambda_2+\lambda_3+\lambda_{12}+\lambda_{13}+\lambda_{23}+\lambda_{123})}{\sum_{\text{LP}}}. \end{aligned} \tag{S1}$$

Case I

Control arm

- $\lambda_1 = \lambda_2 = \lambda_3 = \lambda_\text{c}$
- $\lambda_{12} = \lambda_{13} = \lambda_{23} = 1$
- $\lambda_{123} = 0$

(S1) becomes

$$\exp\,(\lambda_\text{c}) + \exp\,(2\lambda_\text{c}+1) + \frac{1}{3}\exp\,(3\lambda_\text{c}+3) - \frac{\sum_{\text{LP}}}{3}p\,(y=1) = 0.$$

Treatment arm

- $\lambda_1 = \lambda_2 = \lambda_3 = \lambda_\text{t}$

- $\lambda_{12} = \lambda_{13} = \lambda_{23} = 0.5$
- $\lambda_{123} = 0$

(S1) becomes

$$\exp(\lambda_t) + \exp(2\lambda_t + 0.5) + \frac{1}{3}\exp(3\lambda_t + 1.5) - \frac{\sum_{LP}}{3} p(y = 1) = 0.$$

Case II

Control arm

- $\lambda_1 = \lambda_2 = \lambda_3 = \lambda_c$
- $\lambda_{12} = \lambda_{13} = \lambda_{23} = 1$
- $\lambda_{123} = 0.5$

(S1) becomes

$$\exp(\lambda_c) + \exp(2\lambda_c + 1) + \frac{1}{3}\exp(3\lambda_c + 3.5) - \frac{\sum_{LP}}{3} p(y = 1) = 0.$$

Treatment arm

- $\lambda_1 = \lambda_2 = \lambda_3 = \lambda_t$
- $\lambda_{12} = \lambda_{13} = \lambda_{23} = 0.5$
- $\lambda_{123} = 0$

(S1) becomes

$$\exp(\lambda_t) + \exp(2\lambda_t + 0.5) + \frac{1}{3}\exp(3\lambda_t + 1.5) - \frac{\sum_{LP}}{3} p(y = 1) = 0.$$

Case III

Control arm

- $\lambda_1 = \lambda_2 = \lambda_3 = \lambda_c$
- $\lambda_{12} = \lambda_{13} = \lambda_{23} = -\lambda$
- $\lambda_{123} = \lambda$

(S1) becomes

$$\exp(\lambda_c) + \exp(\lambda_c) + \frac{1}{3}\exp(\lambda_c) - \frac{\sum_{LP}}{3} p(y = 1) = 0.$$

Treatment arm

- $\lambda_1 = \lambda_2 = \lambda_3 = \lambda_t$
- $\lambda_{12} = \lambda_{13} = \lambda_{23} = -\lambda_t$
- $\lambda_{123} = \lambda_t$

(S1) becomes

$$\exp(\lambda_t) + \exp(\lambda_t) + \frac{1}{3}\exp(\lambda_t) - \frac{\sum_{LP}}{3} p(y = 1) = 0.$$

S1.1.2 Complex composite endpoint

$$p(y = 0) = \frac{1}{\sum_{LP}} + \frac{\exp(\lambda_1)}{\sum_{LP}} + \frac{\exp(\lambda_2)}{\sum_{LP}} + \frac{\exp(\lambda_3)}{\sum_{LP}} + \frac{\exp(\lambda_2 + \lambda_3 + \lambda_{23})}{\sum_{LP}} \quad \text{(S2)}$$

$$\sum_{LP} = \frac{1 + \exp(\lambda_1) + \exp(\lambda_2) + \exp(\lambda_3) + \exp(\lambda_2 + \lambda_3 + \lambda_{23})}{p(y = 0)}.$$

$$p\left(y=1\right)=\frac{\exp\left(\lambda_1+\lambda_2+\lambda_{12}\right)}{\sum_{\text{LP}}}+\frac{\exp\left(\lambda_1+\lambda_3+\lambda_{13}\right)}{\sum_{\text{LP}}} \qquad \text{(S3)}$$
$$+\frac{\exp\left(\lambda_1+\lambda_2+\lambda_3+\lambda_{12}+\lambda_{13}+\lambda_{23}+\lambda_{123}\right)}{\sum_{\text{LP}}}$$
$$\sum_{\text{LP}}=\frac{\exp\left(\lambda_1+\lambda_2+\lambda_{12}\right)+\exp\left(\lambda_1+\lambda_3+\lambda_{13}\right)+\exp\left(\lambda_1+\lambda_2+\lambda_3+\lambda_{12}+\lambda_{13}+\lambda_{23}+\lambda_{123}\right)}{p\left(y=1\right)}.$$

From (S2) and (S3)

$$\frac{1+\exp\left(\lambda_1\right)+\exp\left(\lambda_2\right)+\exp\left(\lambda_3\right)+\exp\left(\lambda_2+\lambda_3+\lambda_{23}\right)}{p\left(y=0\right)} \qquad \text{(S4)}$$
$$=\frac{\exp\left(\lambda_1+\lambda_2+\lambda_{12}\right)+\exp\left(\lambda_1+\lambda_3+\lambda_{13}\right)+\exp\left(\lambda_1+\lambda_2+\lambda_3+\lambda_{12}+\lambda_{13}+\lambda_{23}+\lambda_{123}\right)}{1-p\left(y=0\right)}.$$

Let $p\left(y=0\right)=p_0$.

<u>Case I</u>

Control arm

- $\lambda_1=\lambda_2=\lambda_3=\lambda_{\text{c}}$
- $\lambda_{12}=\lambda_{13}=\lambda_{23}=1$
- $\lambda_{123}=0$

(S4) becomes

$$(3-3p_0)\exp\left(\lambda_{\text{c}}\right)+(1-3p_0)\exp\left(2\lambda_{\text{c}}+1\right)-p_0\exp\left(3\lambda_{\text{c}}+3\right)+(1-p_0)=0.$$

Treatment arm

- $\lambda_1=\lambda_2=\lambda_3=\lambda_{\text{t}}$
- $\lambda_{12}=\lambda_{13}=\lambda_{23}=0.5$
- $\lambda_{123}=0$

(S4) becomes

$$(3-3p_0)\exp\left(\lambda_{\text{t}}\right)+(1-3p_0)\exp\left(2\lambda_{\text{t}}+0.5\right)-p_0\exp\left(3\lambda_{\text{t}}+1.5\right)+(1-p_0)=0.$$

<u>Case II</u>

Control arm

- $\lambda_1=\lambda_2=\lambda_3=\lambda_{\text{c}}$
- $\lambda_{12}=\lambda_{13}=\lambda_{23}=1$
- $\lambda_{123}=0.5$

(S4) becomes

$$(3-3p_0)\exp\left(\lambda_{\text{c}}\right)+(1-3p_0)\exp\left(2\lambda_{\text{c}}+1\right)-p_0\exp\left(3\lambda_{\text{c}}+3.5\right)+(1-p_0)=0.$$

Treatment arm

- $\lambda_1=\lambda_2=\lambda_3=\lambda_{\text{t}}$
- $\lambda_{12}=\lambda_{13}=\lambda_{23}=0.5$
- $\lambda_{123}=0$

(S4) becomes

$$(3-3p_0)\exp\left(\lambda_{\text{t}}\right)+(1-3p_0)\exp\left(2\lambda_{\text{t}}+0.5\right)-p_0\exp\left(3\lambda_{\text{t}}+1.5\right)+(1-p_0)=0.$$

Case III

Control arm

- $\lambda_1 = \lambda_2 = \lambda_3 = \lambda_\mathrm{c}$
- $\lambda_{12} = \lambda_{13} = \lambda_{23} = 1$
- $\lambda_{123} = 0.5$

(S4) becomes

$$(3 - 3p_0)\exp(\lambda_\mathrm{c}) + (1 - 3p_0)\exp(2\lambda_\mathrm{c} + 1) - p_0\exp(3\lambda_\mathrm{c} + 3.5) + (1 - p_0) = 0.$$

Treatment arm

- $\lambda_1 = \lambda_2 = \lambda_3 = \lambda_\mathrm{t}$
- $\lambda_{12} = \lambda_{13} = \lambda_{23} = 0.5$
- $\lambda_{123} = -0.5$

(S4) becomes

$$(3 - 3p_0)\exp(\lambda_\mathrm{t}) + (1 - 3p_0)\exp(2\lambda_\mathrm{t} + 0.5) - p_0\exp(3\lambda_\mathrm{t} + 1) + (1 - p_0) = 0.$$

Values of λ_c and λ_t were solved for using the community-contributed Stata module `moremata`.[1]

S1.2 Results

S1.2.1 Simple composite endpoint

Performance measures for $\hat{\beta}_0$ are presented graphically in Figures S1–S3.

S1.2.2 Complex composite endpoint

Table S2 summarizes occasions when MI at the component level with imputation stratified by randomized treatment x and fully observed component z_1 (MIC–x–z_1) suffered from perfect prediction. These were handled by using the `augment`[2] option in `mi impute chained`. Performance measures are presented graphically in Figures S4–S6 for $\hat{\beta}_x$, and in Figures S7–S9 for $\hat{\beta}_0$.

S2 Reanalysis of the TOPPS trial

Tables S3 and S4 present supplementary results of the reanalysis of the TOPPS trial.

References

1. Jann B. MOREMATA: Stata module (Mata) to provide various functions. Statistical Software Components S455001, Boston College Department of Economics, revised 06 Dec 2020, 2005.

2. White IR, Daniel R, Royston P. Avoiding bias due to perfect prediction in multiple imputation of incomplete categorical variables. *Computational Statistics and Data Analysis*, 54:2267–2275, 2010.

Table S1 Simulation study: values of the parameters in the log-linear model used to generate data in the components of the composite endpoint

(a) Values of λs chosen for each type of composite endpoint

	Simple composite						Complex composite					
	Case I		Case II		Case III		Case I		Case II		Case III	
λ	$x=0$	$x=1$	$x=0$	$x=1$	$x=0$	$x=1$	$x=0$	$x=1$	$x=0$	$x=1$	$x=0$	$x=1$
λ_1	−1.477	−0.437	−1.538	−0.437	−1.646	−0.296	−0.634	0.977	−0.825	0.977	−0.825	1.297
λ_2	−1.477	−0.437	−1.538	−0.437	−1.646	−0.296	−0.634	0.977	−0.825	0.977	−0.825	1.297
λ_3	−1.477	−0.437	−1.538	−0.437	−1.646	−0.296	−0.634	0.977	−0.825	0.977	−0.825	1.297
λ_{12}	1	0.5	1	0.5	1.646	0.296	1	0.5	1	0.5	1	0.5
λ_{13}	1	0.5	1	0.5	1.646	0.296	1	0.5	1	0.	1	0.5
λ_{23}	1	0.5	1	0.5	1.646	0.	1	0.5	1	0.5	1	0.5
λ_{123}	0	0	0.5	0	−1.646	−0.296	0	0	0.5	0	0.5	−0.5

(b) An example of how the probability of each combination of the components was calculated from the chosen values of λs for a simple composite endpoint (case I). Main effect parameters were constrained to take the same value for simplicity; these parameters were derived from the predefined log odds = 0.3, log odds ratio = 1.35, and two-way and three-way interaction parameters

				$x=0$			$x=1$		
y_{simple}	z_1	z_2	z_3	LP	exp(LP)	$p(z_1, z_2, z_3)$	LP	exp(LP)	$p(z_1, z_2, z_3)$
0	0	0	0	0	1	0.426	0	1	0.161
1	0	0	1	−1.477	0.228	0.097	−0.437	0.646	0.104
1	0	1	0	−1.477	0.228	0.097	−0.437	0.646	0.104
1	0	1	1	−1.953	0.142	0.060	−0.375	0.688	0.111
1	1	0	0	−1.477	0.228	0.097	−0.437	0.646	0.104
1	1	0	1	−1.953	0.142	0.060	−0.375	0.688	0.111
1	1	1	0	−1.953	0.142	0.060	−0.375	0.688	0.111
1	1	1	1	−1.430	0.239	0.102	0.188	1.207	0.194
$y_{\text{simple}}=1$						0.573			0.839

Table S2 Simulation study: number of simulation repetitions n_{rep} (out of N_{rep} = 2 000 repetitions) with perfect prediction when MI at the component level (of a complex composite endpoint) was performed stratified by randomized treatment x and fully observed component z_1. All occurrences of perfect prediction were handled by the augmentation procedure in MI[2]

Data generating mechanism	n_{rep}	%
Case I; MCAR	22	1.1
Case I; MAR1	150	7.5
Case I; MAR2	160	8.0
Case II; MCAR	50	2.5
Case II; MAR1	142	7.1
Case II; MAR2	156	7.8
Case III; MCAR	200	10.0
Case III; MAR1	436	21.8
Case III; MAR2	426	21.3

Table S3 TOPPS reanalysis: number of days (0–5) with a missing bleeding assessment in each of the six time blocks. Proph, Propylactic platelet transfusion arm; No proph: no-Propylactic platelet transfusion arm; $N = 600$

	Block 1			Block 2			Block 3			Block 4			Block 5			Block 6		
No. days	Proph	No proph	$\sum$	Proph	No proph	$\sum$	Proph	No proph	$\sum$	Proph	No proph	$\sum$	Proph	No proph	$\sum$	Proph	No proph	$\sum$
0	291	283	574	279	266	545	274	262	536	282	263	545	285	270	555	283	268	551
%	97.32	94.02	95.67	93.31	88.37	90.83	91.64	87.04	89.33	94.31	87.38	90.83	95.32	89.7	92.5	94.65	89.04	91.83
1	5	11	16	12	19	31	16	15	31	8	12	20	4	3	7	5	6	11
%	1.67	3.65	2.67	4.01	6.31	5.17	5.35	4.98	5.17	2.68	3.99	3.33	1.34	1	1.17	1.67	1.99	1.83
2	2	2	4	4	3	7	3	4	7	1	3	4	3	2	5	2	2	4
%	0.67	0.66	0.67	1.34	1	1.17	1	1.33	1.17	0.33	1	0.67	1	0.66	0.83	0.67	0.66	0.67
3	0	3	3	3	5	8	1	1	2	1	1	2	0	1	1	2	0	2
%	0	1	0.5	1	1.66	1.33	0.33	0.33	0.33	0.33	0.33	0.33	0	0.33	0.17	0.67	0	0.33
4	0	0	0	0	2	2	1	6	7	1	2	3	2	1	3	0	0	0
%	0	0	0	0	0.66	0.33	0.33	1.99	1.17	0.33	0.66	0.5	0.67	0.33	0.5	0	0	0
5	1	2	3	1	6	7	4	13	17	6	20	26	5	24	29	7	25	32
%	0.33	0.66	0.5	0.33	1.99	1.17	1.34	4.32	2.83	2.01	6.64	4.33	1.67	7.97	4.83	2.34	8.31	5.33

Table S4 TOPPS reanalysis: estimated proportions (and associated 95% CIs) of events (in the composite endpoint constructed from six time blocks) by randomized treatment. MIC–trt 1, MI performed by `mi impute chained`, imputation of each block is conditional on all other blocks and stratified by randomized treatment; MIC–trt 2, MI performed by `ice`, imputation of each block is conditional on all other blocks and stratified by randomized treatment; MIC–trt 3, MI performed by `mi impute chained`, imputation of each block is conditional on two adjacent blocks and stratified by randomized treatment. Proph, Propylactic platelet transfusion arm; No proph: no-Propylactic platelet transfusion arm

	Approach 1				Approach 2			
Strategy	Proph	95% CI	No proph	95% CI	Proph	95% CI	No proph	95% CI
CRA	0.430	0.367 to 0.495	0.495	0.427 to 0.564	0.428	0.369 to 0.487	0.493	0.431 to 0.554
Deriv	0.477	0.416 to 0.539	0.564	0.500 to 0.626	0.441	0.383 to 0.501	0.521	0.461 to 0.580
MI–CRA	0.430	0.366 to 0.493	0.496	0.429 to 0.563	0.426	0.368 to 0.484	0.496	0.436 to 0.556
MI–Deriv	0.475	0.414 to 0.537	0.563	0.501 to 0.625	0.441	0.383 to 0.498	0.521	0.464 to 0.579
MIC–main	0.437	0.380 to 0.495	0.507	0.447 to 0.566	0.431	0.375 to 0.488	0.510	0.452 to 0.567
MIC–trt 1					0.432	0.375 to 0.489	0.510	0.452 to 0.567
MIC–trt 2	0.435	0.377 to 0.492	0.497	0.439 to 0.556	0.430	0.374 to 0.487	0.502	0.445 to 0.559
MIC–trt 3	0.438	0.381 to 0.495	0.514	0.455 to 0.573	0.432	0.376 to 0.489	0.509	0.451 to 0.566

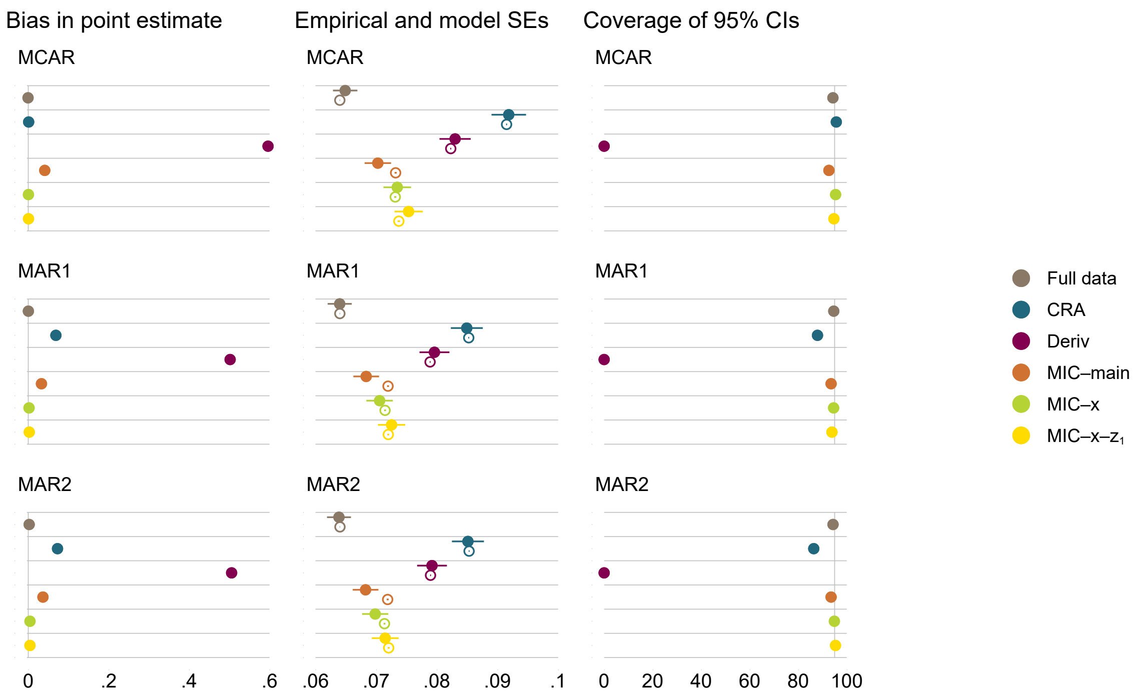

Figure S1 Simple composite endpoint, case I: performance measures for $\hat{\beta}_0$ under different missingness mechanisms of the components; $\beta_0 = 0.3$. Error bars, $\pm 1.96\times$ Monte Carlo errors; filled and hollow points, empirical and average model standard errors, respectively; vertical lines at 0 and 95 for bias and coverage, respectively

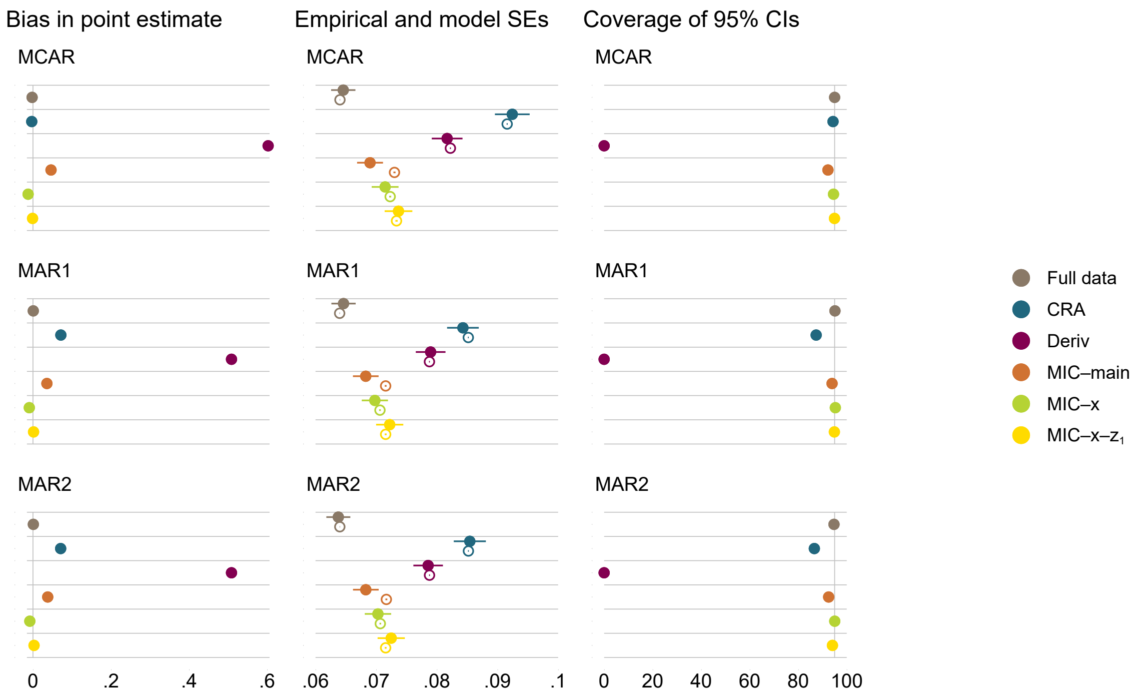

Figure S2 Simple composite endpoint, case II: performance measures for $\hat{\beta}_0$ under different missingness mechanisms of the components; $\beta_0 = 0.3$. Error bars, $\pm 1.96\times$ Monte Carlo errors; filled and hollow points, empirical and average model standard errors, respectively; vertical lines at 0 and 95 for bias and coverage, respectively

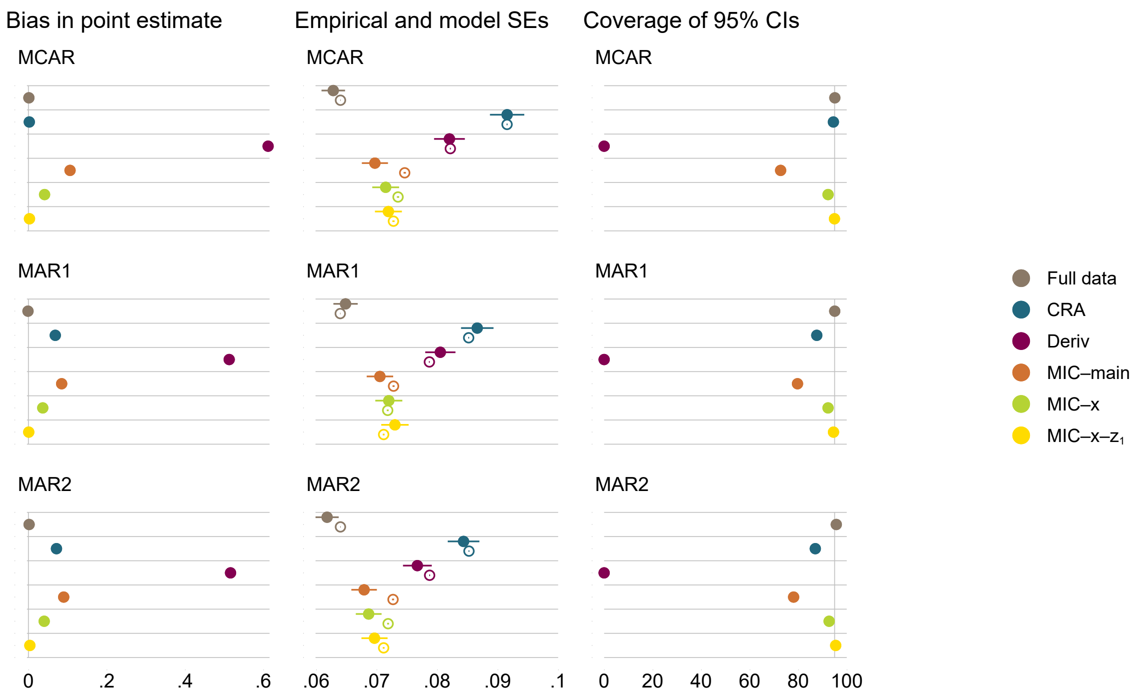

Figure S3 Simple composite endpoint, case III: performance measures for $\hat{\beta}_0$ under different missingness mechanisms of the components; $\beta_0 = 0.3$. Error bars, $\pm 1.96\times$ Monte Carlo errors; filled and hollow points, empirical and average model standard errors, respectively; vertical lines at 0 and 95 for bias and coverage, respectively

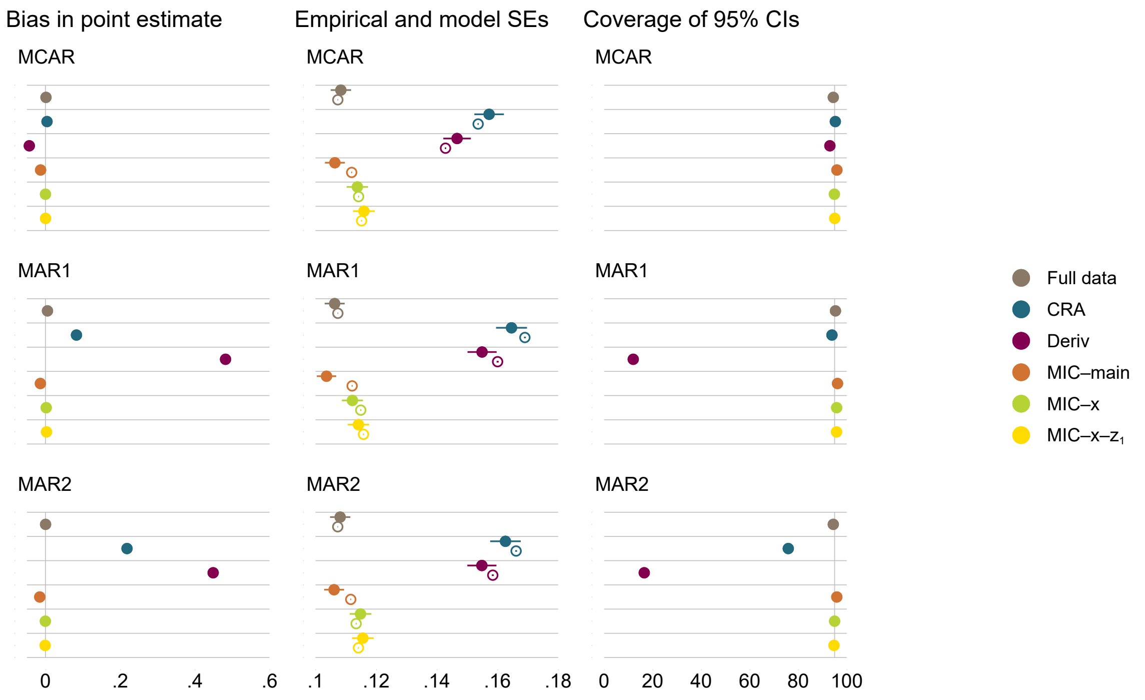

Figure S4 Complex composite endpoint, case I: performance measures for $\hat{\beta}_x$ under different missingness mechanisms of the components; $\beta_x = 1.35$. Error bars, $\pm 1.96\times$ Monte Carlo errors; filled and hollow points, empirical and average model standard errors, respectively; vertical lines at 0 and 95 for bias and coverage, respectively

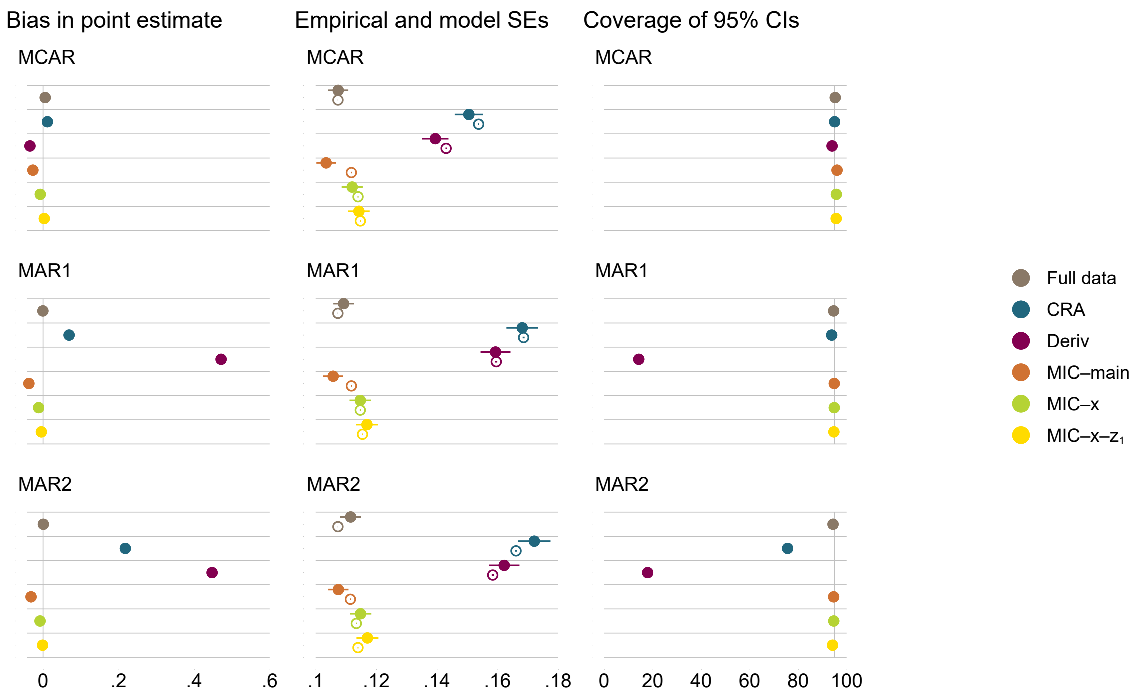

Figure S5 Complex composite endpoint, case II: performance measures for $\hat{\beta}_x$ under different missingness mechanisms of the components; $\beta_x = 1.35$. Error bars, $\pm 1.96\times$ Monte Carlo errors; filled and hollow points, empirical and average model standard errors, respectively; vertical lines at 0 and 95 for bias and coverage, respectively

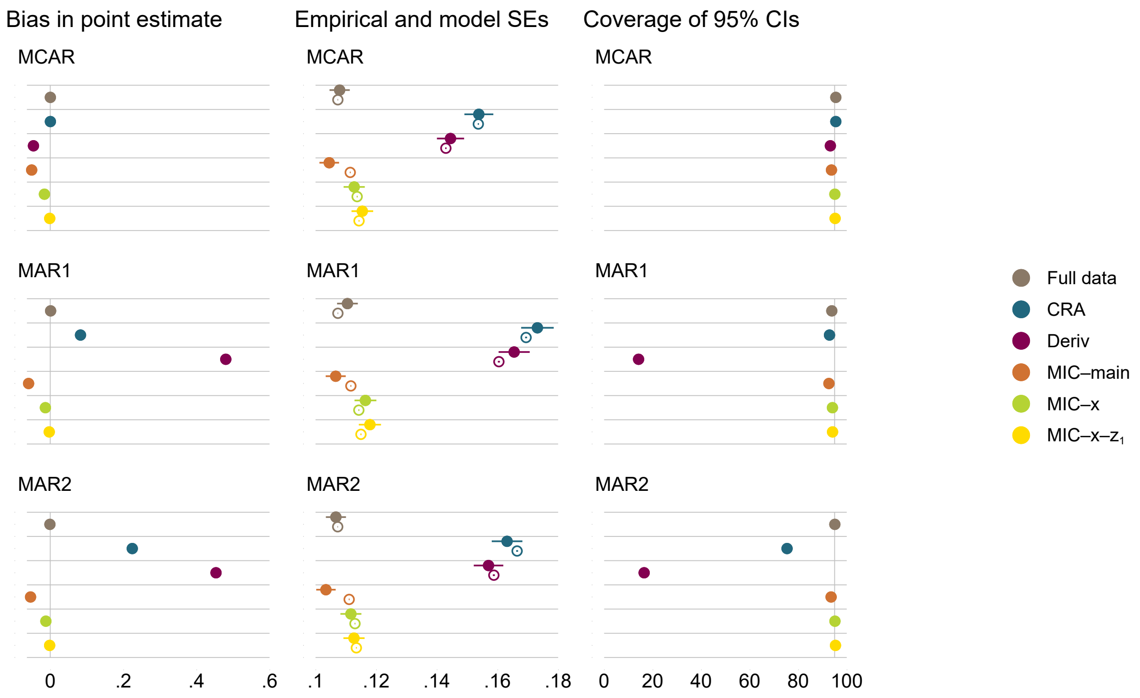

Figure S6 Complex composite endpoint, case III: performance measures for $\hat{\beta}_x$ under different missingness mechanisms of the components; $\beta_x = 1.35$. Error bars, $\pm 1.96\times$ Monte Carlo errors; filled and hollow points, empirical and average model standard errors, respectively; vertical lines at 0 and 95 for bias and coverage, respectively

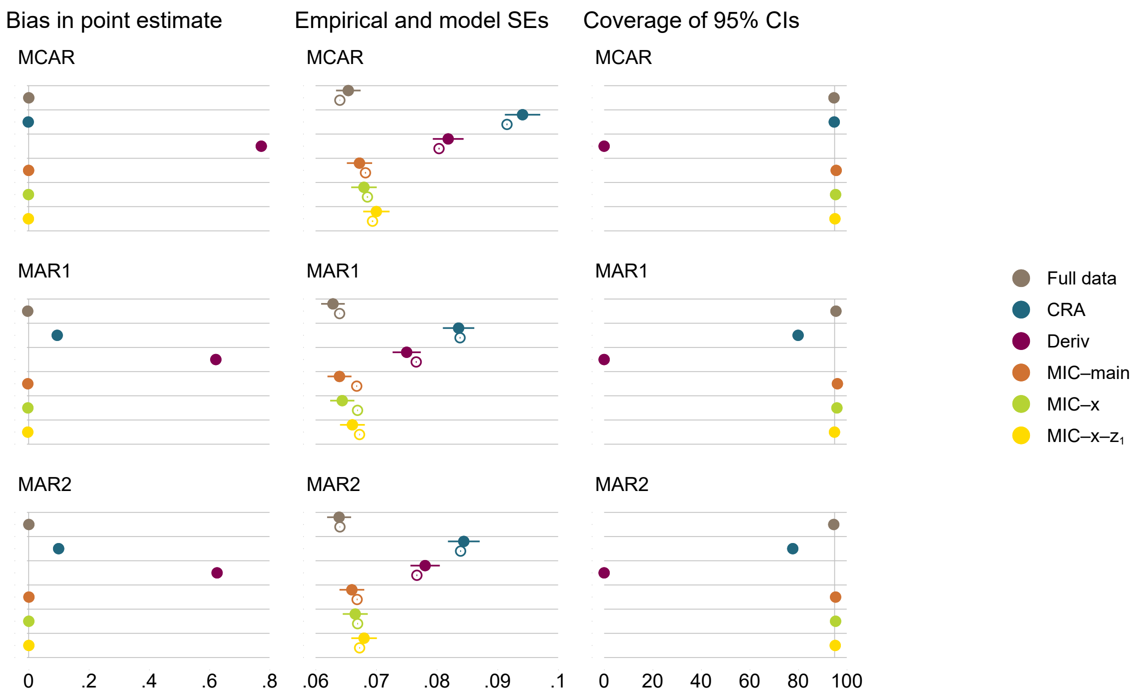

Figure S7 Complex composite endpoint, case I: performance measures for $\hat{\beta}_0$ under different missingness mechanisms of the components; $\beta_0 = 0.3$. Error bars, $\pm 1.96\times$ Monte Carlo errors; filled and hollow points, empirical and average model standard errors, respectively; vertical lines at 0 and 95 for bias and coverage, respectively

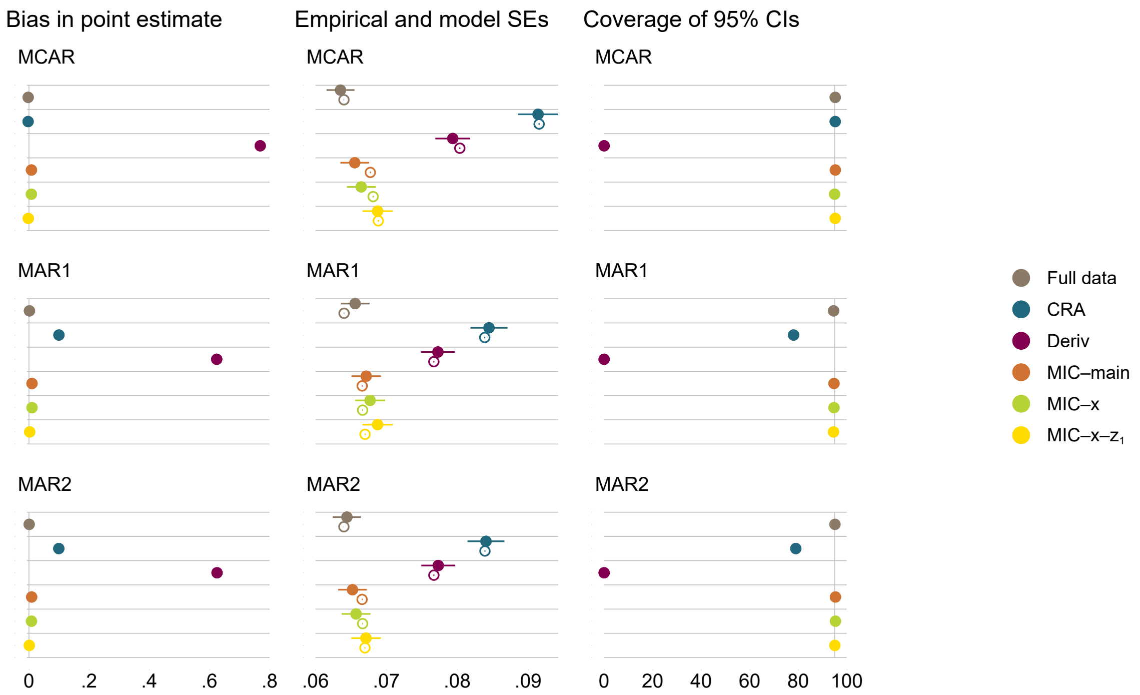

Figure S8 Complex composite endpoint, case II: performance measures for $\hat{\beta}_0$ under different missingness mechanisms of the components; $\beta_0 = 0.3$. Error bars, $\pm 1.96\times$ Monte Carlo errors; filled and hollow points, empirical and average model standard errors, respectively; vertical lines at 0 and 95 for bias and coverage, respectively

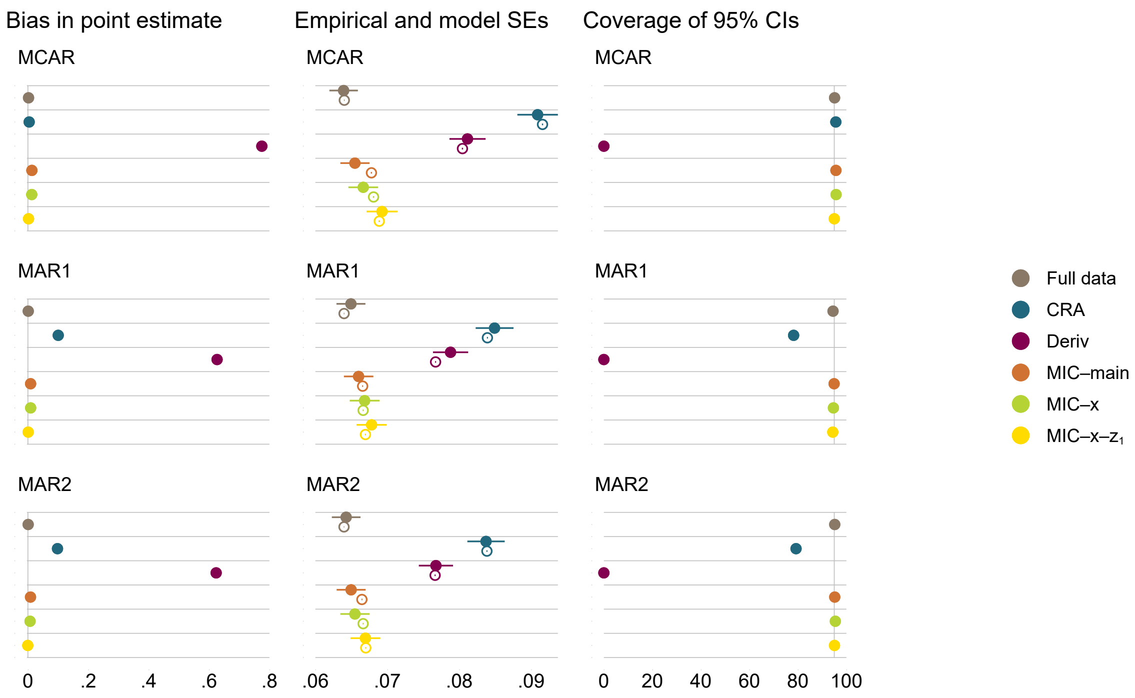

Figure S9 Complex composite endpoint, case III: performance measures for $\hat{\beta}_0$ under different missingness mechanisms of the components; $\beta_0 = 0.3$. Error bars, $\pm 1.96\times$ Monte Carlo errors; filled and hollow points, empirical and average model standard errors, respectively; vertical lines at 0 and 95 for bias and coverage, respectively
